# Epi-nutrients for cancer prevention: Molecular mechanisms and emerging insights

**DOI:** 10.1007/s10565-025-10054-2

**Published:** 2025-07-15

**Authors:** Saad Bakrim, Nasreddine El Omari, Ouadie Mohamed El Yaagoubi, Asaad Khalid, Ashraf N. Abdalla, Siddiqa M. A. Hamza, Salma Elhadi Ibrahim, Farah Atifi, Younes Zaid, Abdelhakim Bouyahya, Meriem El Fessikh, Long Chiau Ming, Tarik Aanniz

**Affiliations:** 1 Geo-Bio-Environment Engineering and Innovation Laboratory, Molecular Engineering, Biotechnology and Innovation Team, Polydisciplinary Faculty of Taroudant, Ibn Zohr University, Agadir, Morocco; 2 High Institute of Nursing Professions and Health Techniques of Tetouan, Tetouan, Morocco; 3 Drug Science Laboratory – Faculty of Medicine, Pharmacy and Dentistry of Fez, Sidi Mohamed Ben Abdellah University, Fez, 30000 Morocco; 4 Health Research Center, Jazan University, P.O. Box: 114, Jazan, Saudi Arabia; 5 Medicinal and Aromatic Plants and Traditional Medicine Research Institute, National Center for Research, P. O. Box 2404, Khartoum, Sudan; 6 Department of Pharmacology and Toxicology, College of Pharmacy, Umm Al-Qura University, Makkah, Saudi Arabia; 7 Department of Physiology, College of Medicine, Umm Al-Qura University, Alqunfudah, Saudi Arabia; 8 Department of Pathology, College of Medicine, Umm Al-Qura University, Alqunfudah, Saudi Arabia; 9 Department of Biology, Immunology and Biodiversity Laboratory, Faculty of Sciences Ain Chock, Hassan II University, Casablanca, Morocco; 10 Department of Biology, Faculty of Sciences, Mohammed V University in Rabat, Rabat, Morocco; 11 Laboratory of Human Pathologies Biology, Faculty of Sciences, Mohammed V University in Rabat, Rabat, Morocco; 12 Datta Meghe College of Pharmacy, Datta Meghe Institute of Higher Education and Research (Deemed to Be University), Sawangi (M), Wardha, India; 13 Faculty of Medical and Life Sciences, Sunway University, Sunway City, Malaysia; 14 Medical Biotechnology Laboratory, Rabat Medical & Pharmacy School, Mohammed V University in Rabat, B.P. 6203 Rabat, Morocco

**Keywords:** Epi-nutrients, Epigenetic modulation, Epigenetic cofactors, Molecular pathways, Cancer prevention

## Abstract

The burgeoning field of epigenetics holds considerable potential in cancer prevention and management, as it targets mechanisms essential to regulating gene expression without altering DNA sequences. Epigenetic processes like DNA methylation, histone modifications, non-coding RNAs, and nucleosome remodelling—play an essential role in cellular differentiation and development, with dysfunction in these pathways often leading to malignancy. Targeting epigenetic regulators, including DNA methyltransferases (DNMTs) and histone deacetylases (HDACs), can suppress cancer cell proliferation, making epigenetics a promising therapeutic frontier. Phytochemicals, natural bioactive compounds predominantly found in vegetables, fruits, and seeds, offer a complementary approach to traditional cancer therapies through their epigenetic influence. These compounds exhibit anti-inflammatory, anti-angiogenic, and antioxidant properties, which modulate pathways and proteins involved in chromatin remodeling and may influence the mammalian epigenome. A diverse spectrum of bioactive dietary ingredients, including curcumin, epigallocatechin-3-gallate (EGCG), genistein, quercetin, resveratrol, and sulforaphane, has gained significant interest for their ability to modulate gene expression and chromatin structure via epigenetic mechanisms. Their potential implications for cancer prevention and their role in regulating key epigenetic genes have been described in numerous investigations. This comprehensive review explores the molecular mechanisms by which dietary bioactive molecules may reverse epigenetic aberrations in cancer cells. It examines the influence of these compounds on DNA methylation, ten-eleven translocation (TET) enzymes, and histone modifications, while discussing their specific molecular targets in various cancer types. Additionally, we highlight the pathways through which these epi-nutrients may impact gene expression and enzyme activities associated with epigenetic regulation, which leads to innovative, diet-based anticancer strategies. Clinical trial number: not applicable.

## Introduction

Tumor heterogeneity remains a significant challenge in developing effective cancer therapies, even as clinical evidence of the benefits of cancer drugs continues to grow (Wu et al. 2024). Understanding the origins of tumor variability has advanced with high-throughput screening technologies, revealing that heterogeneity can arise not only from intrinsic tumor traits but also from the surrounding microenvironment. This variability fosters tumor growth and diminishes treatment efficacy (Imodoye et al. 2024). Indeed, cancer progression is marked by characteristics such as sustained proliferative signaling, evasion of growth suppressors, resistance to apoptosis, angiogenesis, and metastasis, as well as inflammation and genomic instability (Hanahan and Weinberg 2017). Although cancer was historically considered a genetic disease, recent insights underscore its complexity, with epigenetic dysregulation emerging as a key factor in cancer development (Babar et al. 2022; El Omari et al. 2021a, b).

Epigenetic mechanisms—such as DNA methylation, histone modifications, and non-coding RNA interactions—are fundamental to gene regulation and can contribute to oncogene activation or tumor suppressor gene (TSG) silencing (Bouyahya et al. 2022a; Ramaiah et al. 2021). Unlike genetic mutations, which alter the DNA sequence, epigenetic changes impact chromatin structure and gene expression without modifying the DNA itself (Schulz 2023). Cancer initiation is often accompanied by epigenetic changes affecting cancer-related genes, with DNA methylation, RNA modifications, and histone alterations identified as central players (Casali et al. 2024). Notably, the reversibility of these epigenetic modifications makes them promising therapeutic targets for enhancing the effectiveness of standard cancer drugs (Carlos-Reyes et al. 2019).

In addition to pharmacologic therapies, natural compounds in dietary sources have garnered attention for their potential role in cancer management. Phytomedicine is widely used across cultures, with natural compounds increasingly recognized as modulators of epigenetic pathways (Jazieh et al. 2021). Exploring plant-derived bioactives constitutes a transformative area of medicinal chemistry, especially due to their potential to modulate epigenetic pathways associated with histone modifications and DNA methylation. Such substances as EGCG, curcumin, resveratrol, and genistein have shown their capacity to reverse abnormal epigenetic alterations linked to the activation of oncogenes and the inhibition of tumor suppressor genes. These characteristics highlight their suitability as valuable agents for cancer prevention and therapy, often gaining significant interest beyond clinical use (Fatima et al. 2021).

Diet significantly influences health, with inadequate nutrition linked to chronic diseases such as type 2 diabetes mellitus (T2DM), cancer, obesity, cardiovascular disease (CVD), and neurodegenerative disorders (Martino et al. 2024). Phytochemicals, such as curcumin, epigallocatechin-3-gallate (EGCG), genistein, quercetin, resveratrol, and sulforaphane, have shown promise in modulating cancer-associated epigenetic processes like DNA methylation, histone modification, and non-coding RNA regulation (Andreescu et al. 2018; Bakrim et al. 2023; Paluszczak et al. 2010).

These bioactive compounds can alter cellular functions relevant to cancer, including proliferation, invasion, metastasis, and cell death, by modulating both oncogenes and TSGs. Phytochemicals are particularly intriguing as they may inhibit key epigenetic enzymes such as histone deacetylases (HDACs) and DNA methyltransferases (DNMTs) (Aanniz et al. 2024; Bouyahya et al. 2022b; Sailo et al. 2024). Studies have identified various phytochemicals as inhibitors of HDACs and DNMTs, impacting apoptosis, cell cycle arrest, DNA repair, and angiogenesis, all critical pathways in cancer prevention and treatment (Duthie 2011a; Ho et al. 2011; Mikkelsen et al. 2021). Additionally, essential micronutrients in the diet, such as vitamins and minerals, serve as cofactors for enzymes and play critical roles in epigenetic regulation. In the absence of these cofactors, enzymes cannot function as intended or be properly active (Singh et al. 2020). In fact, vitamin C affects TET enzyme activity, influencing epigenetically regulated gene expression (Hore et al. 2016; Mastrangelo et al. 2018). Similarly, selenium promotes DNA repair and cell cycle regulation (Tiffon 2018), while other nutrients, like choline, folate, vitamin B12, and zinc, support DNMT function and DNA methylation stability (Allison et al. 2021).

By serving as activators, inhibitors, or substrates for epigenetic enzymes, dietary components underscore the connection between nutrition and epigenetic regulation (Reid et al. 2017). The capacity of phytochemicals to modulate epigenetic mechanisms represents an innovative therapeutic avenue with potential for new biomarkers and preventive strategies in cancer.

This review synthesizes recent findings on dietary bioactive compounds that reverse abnormal epigenetic changes in cancer cells, focusing on key mechanisms such as DNA methylation, TET enzymes, and histone modifications. It also highlights their molecular targets in different types of cancer and investigates how these natural compounds can influence gene expression or modify enzyme activity, which is helpful in developing diet-based cancer prevention strategies. The studies analyzed, mainly covering the period from 2015 to 2025, represent current progress in this field of research. Studies were selected based on their scientific relevance, clarity of molecular mechanisms, and ability to enhance our knowledge of nutrient-mediated epigenetic regulation, particularly concerning cancer. Only a limited number of previous references providing a fundamental understanding of underlying epigenetic processes, such as DNA methylation and chromatin remodeling, were included.

## Gene expression as a fundamental driver in cancer progression

Tumorigenesis is a multifaceted, multistage process transforming normal cells into malignant ones, primarily by disrupting normal cellular regulatory pathways and promoting unchecked cell division. Cancer is characterized by hallmark traits that distinguish it from healthy tissue, including sustained proliferative signaling, evasion of growth suppressors, resistance to programmed cell death (apoptosis), limitless replicative potential, induction of angiogenesis, capacity for invasion and metastasis, genomic instability, immune evasion, and dysregulated energy metabolism (Hanahan and Weinberg 2017).

Initially perceived as a solely genetic disorder, cancer is now recognized as a complex multifactorial disease, encompassing somatic and inherited genetic mutations as well as epigenetic dysregulation, which plays a significant role in tumor development and progression (Babar et al. 2022; El Omari et al. 2021a, b; Feng and De Carvalho 2022).

For instance, *Kirsten rat sarcoma viral oncogene homolog* (*KRAS*) is one of the most frequently mutated proto-oncogenes found in cancers and encodes a small GTPase belonging to the RAS protein family. An estimated 20% of all human cancers present mutations in this gene, with especially high frequencies in pancreatic (88%), colorectal (50%), and lung (32%) cancers (Casacuberta-Serra et al. 2024). In fact, the observed variants are usually missense mutations such as G12D and G12V (both corresponding to rs121913529), as well as G12R and p.G12C (both corresponding to rs121913530) (Bannoura et al. 2022; Malhotra et al. 2024; Zhu et al. 2021). These variants subsequently cause a RAS-protein gain of function associated with enhanced GTP activity, since GTPase-activating proteins (GAPs) are unable to bind KRAS, thus increasing the intrinsic GTPase activity of KRAS (Johnson et al. 2022). Due to its involvement in the control of several important cellular processes, *KRAS* mutations lead to disruption of normal cell cycle progression and aberrant proliferation through abnormal activation of signaling pathways such as mitogen-activated protein kinase (MAPK) and phosphoinositide 3-kinase (PI3K) (Lam et al. 2022; Zhu et al. 2021).

Besides, the tumor suppressor gene, *p53* (*TP53*), encodes a transcription factor that regulates the gene expression involved in signaling pathways and regulates cell cycle, DNA repair, and apoptosis (Toufektchan and Toledo 2018). Deregulation of the p53 pathway causes its contribution to nearly half of human cancers in pathogenesis, including ovarian, colon and rectal, lung, pancreatic, stomach, urethral, liver, breast, and prostate cancers (Wang et al. 2023; Zhou et al. 2019). Approximately 80% of the variants identified in the *p53* gene are point mutations situated in a few hotspot codons and resulting in the loss of the tumor suppressor function and a dominant-negative effect of p53 activity, along with a gain of new oncogenic functions leading to therapeutic resistance (Giacomelli et al. 2018; Zhou et al. 2019). These alterations occur in particular in the central DNA-binding domain (DBD) and are classified as mutations affecting DNA contact, as a result of changes of critical amino acids indispensable for p53 binding, such as R248Q (rs11540652), R273H (rs28934576), and R282W (rs28934574), or conformational mutations that lead to either a loss of protein structure or altered conformation, such as R175H (rs28934578), Y220C (rs121912666), G245S (rs28934575), and R249S (rs28934571) (Zhou et al. 2019). Genes repressed by wild-type p53, such as *cyclin-dependent kinase 1* (*CDK1*)*, cyclin-A2* (*CCNA2*), and *cyclin B1/2* (*CCNB1/2*), become aberrantly activated in the case of *p53* mutations (R175H, R273H, and D281G), enabling cells to escape the control of cell proliferation and progress towards cancerous transformation. In contrast, *p53* mutations might promote activation of proto-oncogenes such as the *MYC* gene that amplifies cancer cell proliferation (Chiang et al. 2021; Hernández Borrero and El-Deiry 2021). Furthermore, mutations in the *p53* gene are also associated with genomic instability due to impaired DNA damage repair mechanisms (Liu, Song, and Xu 2010). The R248W and R273H mutations attribute Mre11 nuclease-binding capacity to the p53 protein to prevent association of the Mre11-Rad50-NBS1 (MRN) complex with DNA double-strand breaks, thereby impairing ataxia-telangiectasia mutated (ATM) activation and the DNA damage response (Calheiros et al. 2023; Eischen 2016). ATM is indeed a protein belonging to the phosphatidylinositol 3-kinase-related kinases (PIKKs) family and is a major activator involved in the cellular response to DNA double-strand breaks, genomic stability, regulation of cell cycle, and cell survival (Marechal and Zou 2013). Moreover, it promotes DNA repair by phosphorylating histone H2AX and activating the G1/S checkpoint after phosphorylation of p53 (Lee 2024). Reported mutations in the *ATM* gene usually involve small deletions and insertions as well as single-nucleotide polymorphisms and lead to a complete absence or impaired function of the protein, mainly observed in sporadic breast cancer and hematological malignancies like T-cell prolymphocytic leukemia and mantle cell lymphoma (Lee 2024).

The pathogenesis of cancer involves critical genes, such as proto-oncogenes, TSGs, and DNA repair genes, which function as key regulatory components in cellular processes: (i) Proto-oncogenes encode proteins essential for cell growth, proliferation, and differentiation, including growth factors, receptors, and signal transducers. (ii) TSGs, or “gatekeepers,” oversee cell cycle regulation, apoptosis, and growth suppression and are instrumental in maintaining cellular homeostasis. (iii) DNA repair genes, referred to as “caretakers,” are vital in identifying and repairing DNA damage, thereby preserving genomic stability (Bouyahya et al. 2022a; Ramaiah et al. 2021).

## Epigenetics as a mechanism of gene expression regulation in cancer

Epigenetics encompasses reversible modifications to gene expression without changes in the DNA sequence itself (Cheng et al. 2019; Jenke et al. 2021). The paradigm of cancer development now emphasizes that carcinogenesis is a result of cumulative genetic and epigenetic changes that collectively endow tumors with traits of adaptability and heterogeneity (Lu et al. 2020; Mancarella and Plass 2021). Key epigenetic mechanisms include DNA methylation, histone modifications, non-coding RNAs, and nucleosome remodeling, which work in concert to alter chromatin structure and regulate gene expression in cancer (Bouyahya et al. 2022b; El Omari et al. 2021b).

For instance, aberrant DNA methylation of the *mutL homolog 1* (*MLH1*) promoter can induce the inactivation of this gene, thus leading to a mismatch DNA repair (MMR) pathway disturbance in 20% of colorectal cancer and resulting in microsatellite instability (MSI) (Geissler et al. 2024). Moreover, the epigenetic modifier enhancer of zeste homolog (EZH2) overexpression in breast cancer results in the trimethylation of histone H3 at lysine 27 (H3K27me3), a process that occurs at the promoter of *programmed death ligand 1 (PD-L1)* and causes the inhibition of the immune response-related genes and the enhancement of drug resistance and metastatic capacity (Y. Yang et al. 2022). In certain rare ovarian cancers, mutations have been identified in the *SMARCA4* and *SMARCA2* genes, which encode ATPases of the SWI/SNF (switch/sucrose non-fermentable) chromatin remodeling complex. This complex controls chromatin accessibility to factors involved in DNA repair and gene expression. Mutations in *SMARCA4* and *SMARCA2* genes, therefore, affect chromatin accessibility and the expression of genes linked to differentiation, DNA repair, and epithelial-mesenchymal transition (EMT), ultimately disrupting epigenetic regulation and promoting tumor progression (Ma et al. 2024).

**Fig. 1:**
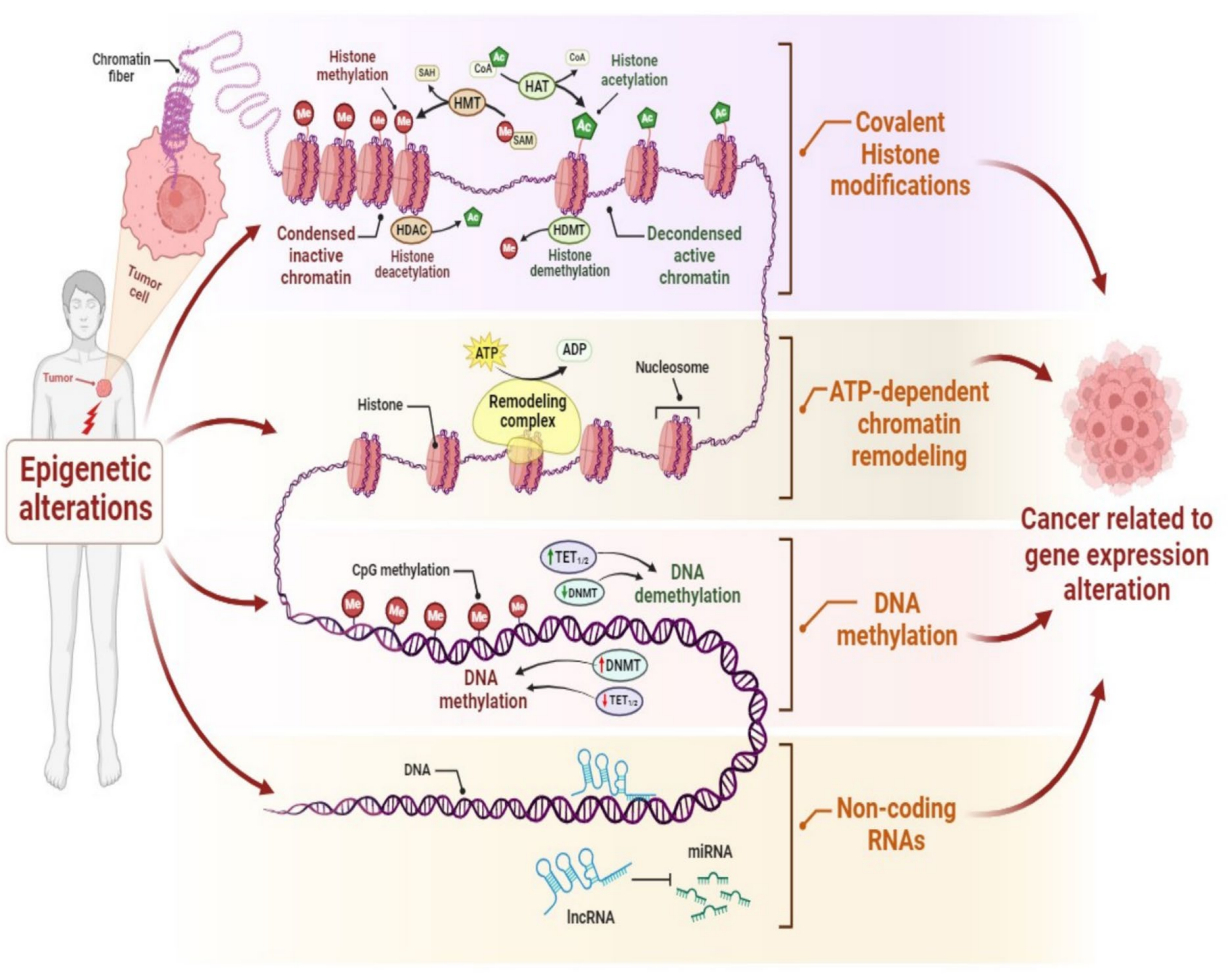
Genomic mechanisms underlying epigenetic alterations in cancer diseases. The human cell needs strategies to control its transcriptome, which maintains the essential DNA sequence. These processes control the epigenetic and epitranscriptomic states of RNA and DNA, respectively. These alterations manifest through modifications of mRNA, nucleosome remodeling, non-encoding RNAs, DNA methylation, and histone modifications. The onset and progression of cancer can be attributed, in part, to the breakdown of epigenetic regulation within the cell. ***Abbreviations:*** DNMT, DNA methyltransferase; HDAC, histone deacetylase; HMT, histone methyltransferase; SAM, S-adenosylmethionine; SAH, S-adenosylhomocysteine; HAT, histone acetyltransferase; TET1/2, ten-eleven translocation methylcytosine dioxygenase 1/2; miRNA, microRNA; lncRNA, long noncoding RNA

Similarly, the non-coding RNAs, such as microRNAs (miRNAs) and long non-coding RNAs (lncRNAs), regulate biological processes, and their dysregulation by epigenetic mutations plays a crucial role in cancer development (Ma et al. 2023). Indeed, miRNAs regulate the expression of target genes at the post-transcriptional level, thus regulating cell growth, division, and apoptosis (Prabhakaran et al. 2024). By binding to the complementary sequence, miRNAs interact with mRNAs, inducing their degradation or inhibiting protein synthesis. LncRNAs are also involved in the regulation of cancer-related gene expression by forming a complex with miRNAs (Ma et al. 2023; Prabhakaran et al. 2024). In hepatocarcinoma, anti-apoptotic miRNAs like miR-21, miR-221/222, and miR-155 are overexpressed, leading to the downregulation of tumor suppressor genes like *phosphatase and tensin homolog deleted on chromosome ten* (*PTEN*), *cyclin-dependent kinase inhibitor 1B* (*DKN1B)/p27*, *cyclin-dependent kinase inhibitor 1C* (*CDKN1C*)/*p57*, and *toll-like receptor 3 (TLR3)*. On the other hand, pro-apoptotic miRNAs, like the let-7 and miR-15 families, are downregulated, thus inhibiting apoptosis and promoting tumor progression (Mahboobnia et al. 2024; Morishita et al. 2021). Besides, abnormal expression of lncRNA RMST is detected in triple-negative breast cancer (TNBC) and plays an important role in tumor cell proliferation and migration. Indeed, this reduction in lncRNA RMST prevents the inhibition of miRNA (miR-4295) on *inositol-1,4,5-trisphosphate receptor type 1* (*ITPR1*) mRNA and involves down-regulated expression of the ITPR1 protein, inducing inhibition of apoptosis and autophagy (Zhang et al. 2025). Notably, these genetic and epigenetic pathways interact, establishing cancer as a disease influenced by both intrinsic and extrinsic factors rather than isolated genetic events (Ramaiah et al. 2021) (Fig. 1).

Epigenetic regulators include “writers,” which add epigenetic marks (e.g., DNMTs, histone acetyltransferases [HATs], and histone methyltransferases [HMTs]); “erasers,” which remove these marks (e.g., HDACs); “readers,” which recognize specific epigenetic marks and relay regulatory signals; and “remodelers,” which modify chromatin accessibility to influence gene expression (Babar et al. 2022; Yang et al. 2016).

Recent studies have underscored the role of the TET family of enzymes, a new class of epigenetic regulators that catalyze the conversion of 5-methylcytosine (5-mC) to 5-hydroxymethylcytosine (5-hmC), thereby facilitating DNA demethylation and impacting gene expression (Babar et al. 2022). Dysregulation in any of these epigenetic modulators can contribute to the loss of cellular memory, potentially leading to carcinogenesis.

## Factors influencing gene expression through epigenetic modifications

Epigenetic dysregulation is a key factor in several stages of tumorigenesis. For example, global DNA hypomethylation can lead to genomic instability, facilitating cancer initiation. Simultaneously, promoters of tumor suppressor genes, notably cyclin-dependent kinase inhibitor *p16INK4a* and MutL Homolog 1 (*MLH1*), are silenced by hypermethylation, accelerating tumor progression and invasion (Wettergren et al. 2025). Moreover, abnormal histone changes and altered expression of non-coding RNAs are other factors leading to metastasis and resistance to treatment (Yu et al. 2024).

As early changes that disrupt cellular homeostasis and promote genomic instability, epigenetic modifications are now considered “first hits” in tumorigenesis, particularly through the altered expression of TSGs, which are often epigenetically silenced rather than mutated (Bouyahya et al. 2022a).

The most recognized epigenetic alteration in cancer is hypermethylation of CpG islands (CGIs) in gene promoters, leading to TSG silencing. On the other hand, hypomethylation of oncogenes, such as *retinoblastoma binding protein 6* (*RBBP6*) (Xiao et al. 2019), *solute carrier family 34 member 2* (*SLC34A2*) (He et al. 2020), and *lymphocyte antigen 6 complex locus K* (*LY6K*) (Sastry et al. 2020), is frequently observed in numerous cancers, facilitating aberrant gene activation. Repressive histone modifications, such as histone hypoacetylation and hyperacetylation, further contribute to TSG silencing (Fahrner et al. 2002).

Emerging evidence points to both programmed transcriptional alterations and stochastic dysregulation of epigenetic modifiers, like DNMTs, HDACs, TET enzymes, and EZH2, as sources of aberrant epigenetic changes that drive oncogenesis (Blanco et al. 2020; Jenke et al. 2021). Understanding how these factors disrupt gene expression offers knowledge about their potential as therapeutic targets, allowing for innovative strategies in cancer prevention and treatment by targeting the epigenome.

### Enzymes

Epigenetic regulation in cancer involves several critical enzymes that alter gene expression through modifications of DNA and histone proteins. Among these enzymes, DNMTs, HATs, HDACs, and TET enzymes play pivotal roles by impacting the DNA methylation and histone acetylation states, which are integral to transcriptional control. This section explains their roles, mechanisms, and implications in cancer.

#### DNA Methyltransferases (DNMTs)

DNMTs catalyze the conversion of cytosine to 5-mC at CpG dinucleotides, a critical process for establishing and maintaining DNA methylation patterns. These enzymes are categorized into maintenance methyltransferases (DNMT1) and de novo methyltransferases (DNMT3A and DNMT3B), each serving distinct roles in cellular methylation dynamics. DNMT1 maintains established methylation patterns during DNA replication, ensuring stable gene repression across cell divisions, while DNMT3A and DNMT3B facilitate de novo methylation, which is crucial during early development and cellular differentiation (Jin and Liu 2018; Mirza et al. 2013).

The dysregulation of DNMTs is associated with aberrant methylation patterns in cancer. Hyperactivity of DNMTs leads to hypermethylation, particularly at promoter CpG islands of TSGs, silencing their expression and thereby promoting tumorigenesis (Jin and Liu 2018; Mirza et al. 2013). Conversely, hypomethylation due to low DNMT activity can activate oncogenes by reducing DNA methylation at gene bodies and repetitive elements, destabilizing the genome (Ehrlich 2009; Van Tongelen et al. 2017). Such dysregulation fosters a permissive environment for malignant transformation by altering gene expression networks essential for cellular growth control, apoptosis, and DNA repair.

Studies further suggest that DNMT inhibitors, targeting aberrant DNMT activity, offer therapeutic potential in cancer treatment by reactivating silenced TSGs and restoring normal gene expression profiles (Van Tongelen et al. 2017).

#### Histone Acetyltransferases (HATs) and Histone Deacetylases (HDACs)

Histone acetylation, mediated by HATs, and deacetylation, controlled by HDACs, are critical for chromatin structure modulation and gene regulation. Acetylation of histones neutralizes the positive charge on histone tails, reducing their interaction with negatively charged DNA, thus promoting a more open chromatin structure conducive to gene transcription (Shechter et al. 2007). Conversely, HDACs remove acetyl groups, condensing chromatin structure and repressing gene transcription (Ropero and Esteller 2007).

The balance between HATs and HDACs is vital for normal gene expression, and disruptions in this balance are frequently observed in cancer. HDACs are organized into four families, encompassing 18 isoforms that interact with key transcription factors such as retinoblastoma protein (RB), transcription factor II E (TFIIE), signal transducer and activator of transcription 3 (STAT3), E2F, p53, and nuclear factor kappa B (NF-κB) (Lin et al. 2006). Through these interactions, HDACs directly influence gene expression patterns, cell cycle progression, apoptosis, and DNA repair mechanisms.

HDAC overexpression in cancer leads to the silencing of TSGs, contributing to uncontrolled cell proliferation and resistance to apoptosis. HDAC inhibitors have thus emerged as promising anti-cancer agents, capable of reactivating TSGs and sensitizing cancer cells to apoptosis and immune recognition by restoring proper acetylation balance (Bouyahya et al. 2022b).

#### Ten-Eleven Translocation (TET) Enzymes

DNA methylation profoundly influences genomic integrity, transcriptional mechanisms, and typical developmental processes. As previously discussed, DNMTs facilitate the methylation of cytosine to 5mC, which functions as a transcriptional repressor and consequently modulates gene expression (Aanniz et al. 2024; Ficz and Gribben, 2014; Vasanthakumar and Godley, 2015; M. Yu et al. 2012). The TET family of proteins (TET1, TET2, and TET3) plays a key role in active DNA demethylation by catalyzing the conversion of 5-mC to 5-hmC, a process critical for reactivating silenced genes and sustaining gene expression essential for cellular development (Cheng et al. 2019; Hu et al. 2015). The discovery of TET enzymes is regarded as a significant advancement in comprehending epigenetic DNA changes (Delhommeau et al. 2009; Ferrone et al. 2020; S. Jiang, 2020; Joshi et al. 2022; Lazarenkov and Sardina, 2022; Lio et al. 2019; López-Moyado et al. 2024; Nickerson et al. 2017; Sasidharan Nair et al. 2018; Singh et al. 2020; D. Zhang et al. 2019). Originally defined by their capacity to oxidize 5mC to 5hmC, it is now apparent that these enzymes also promote further processes, culminating in active DNA demethylation, with their dysregulation affecting cancer-related genes implicated in migration, invasion, and apoptosis (Salmerón-Bárcenas et al. 2023; Zacapala-Gómez et al. 2024). Furthermore, their activity is crucial for the regulation of epigenetic memory and cellular identity (X. Zhang et al. 2023).

**Fig. 2:**
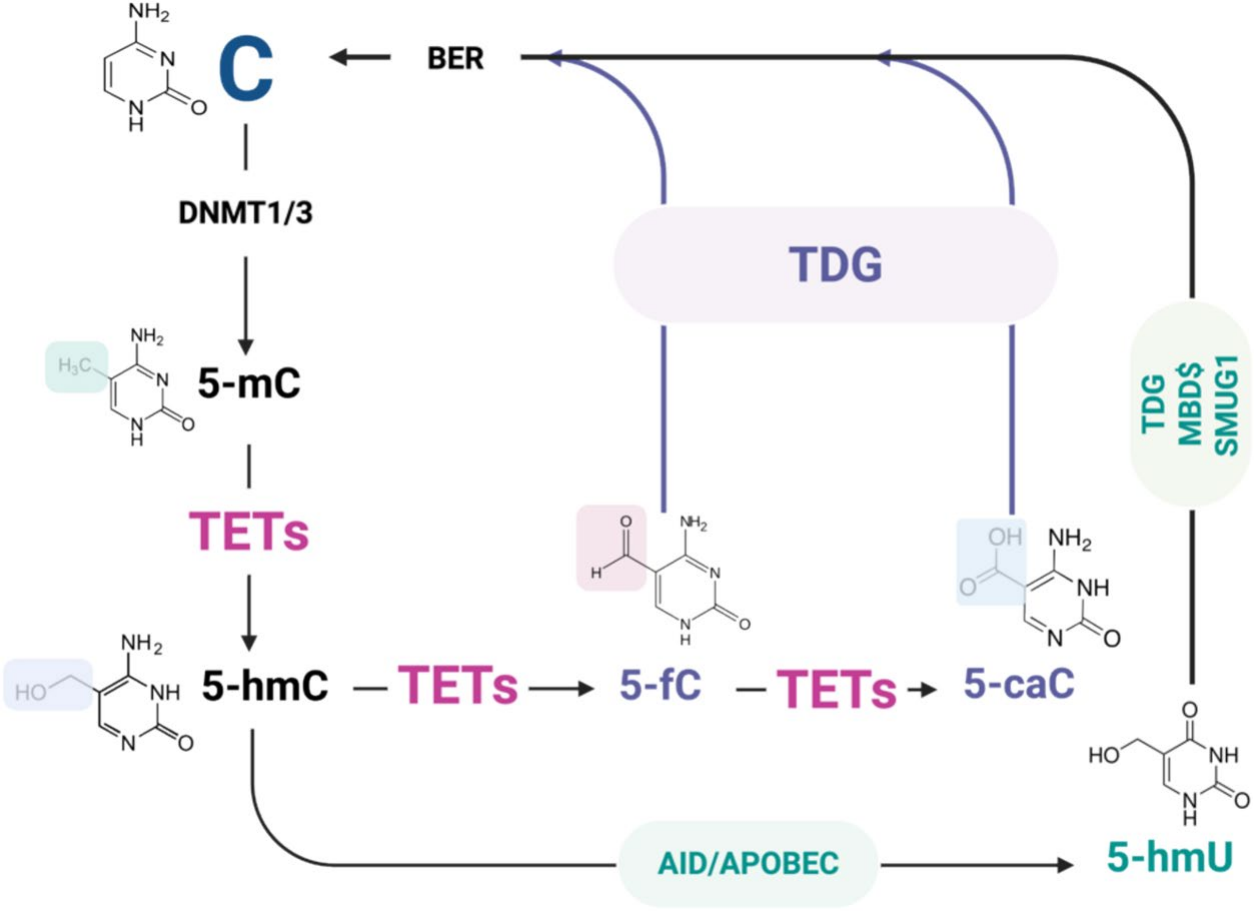
TET enzymes and DNA demethylation: TET family proteins oxidize 5-methylcytosine (5mC) into 5-hydroxymethylcytosine (5hmC), 5-formylcytosine (5fC), and 5-carboxylcytosine (5caC). The 5-fC and 5-caC are recognized and excised by Thymine DNA Glycosylase (TDG). The 5-hmC undergoes deamination to 5-hmU via Activation-Induced Deaminase (AID) proteins, a member of the AID and apolipoprotein B mRNA editing enzyme, catalytic polypeptide (APOBECs) family, and is then excised by TDG, potentially in association with MBD4 and SMUG1 (a monofunctional glycosylase selective for single-stranded uracil-containing DNA). The resulting abasic sites, following the excision of modified bases, are ultimately repaired and replaced by unmodified cytosine through a mechanism involving the Base Excision Repair (BER) pathway

DNA demethylation can occur through the substitution of TET enzyme byproducts, 5fC and 5caC, with cytosine via replication-dependent dilution (passive demethylation) or through active demethylation facilitated by thymine DNA glycosylase (TDG) and base excision repair (BER) processes (Aanniz et al. 2024; Maiti and Drohat, 2011; Smith and Meissner, 2013). TDG can cleave the glycosidic bond between the abasic site and 2-deoxyribose, thereby triggering BER mechanisms that generate abasic sites. These are later repaired with newly synthesized DNA, substituting the corresponding nucleoside opposite the abasic site, ultimately resulting in the loss of 5mC and passive DNA demethylation (Fig. 2). This process, alongside DNA repair mechanisms like BER or mismatch repair (MMR), produces demethylated derivatives of 5mC, including 5-hydroxymethyluracil (5hmU), 5-formyluracil (5fU), and 5-carboxyuracil (5caU). Subsequently, they can be further processed by TDG to regenerate cytosine (Joshi et al. 2022; Palit et al. 2023; Yin et al. 2022; X. Zhang et al. 2023).

Aberrant DNA methylation correlates with dysregulated gene expression in cancer, and TET proteins are frequently inactivated in numerous malignancies by genetic mutations, translocations, deletions, or promoter hypermethylation (Gerecke et al. 2022). TET enzymes and their oxidized product, 5hmC, are also pivotal in recruiting transcription factors and other regulatory proteins, hence impacting cancer progression (Izzo et al. 2021). TET1 is often expressed in embryonic stem cells (ESCs) and downregulated in differentiated tissues, while the loss of its expression causes a global decrease in 5hmC levels (Cimmino et al. 2017). TET2 is broadly expressed in hematopoietic (blood) cells and some other tissues, but especially in hematopoietic stem cells (HSCs), ensuring a major role in blood cell differentiation (Solary et al. 2014). TET3 remains the highly expressed TET member in oocytes and zygotes (Arand et al. 2022).

Overexpression of TET1 and TET2 has been shown to result in a global reduction in 5-mC, suggesting their vital role in regulating DNA methylation dynamics (Jenke et al. 2021). Mutations in *TET1* or *TET2* have been proposed to lead to widespread epigenetic alterations; however, it remains uncertain whether these mutations alone are sufficient to establish a cancer-specific epigenetic signature (Mancarella and Plass 2021).

Unique chromosomal translocations involving *TET1* and *mixed lineage leukemia* (*MLL*) have been identified in certain cancers, further implicating TET proteins in oncogenic pathways (Huang et al. 2013). TET2 remains the most frequently mutated or deleted TET member in a variety of hematological malignancies, and approximately 15% of individuals with myeloid malignancies harbor a mutation or deficiency in *TET2*, which correlates with disease progression (Delhommeau et al. 2009). Reduced expression of TET proteins has been associated with significant decreases in 5-hmC levels across multiple cancer types, including breast, liver, lung, pancreatic, and prostate cancers, suggesting a tumor-suppressive role (Yang et al. 2013). Specifically, TET1 deficiency has been linked to increased tumor cell invasion and enhanced xenograft tumor growth in breast and prostate cancer models, highlighting its potential as a therapeutic target (Cheng et al. 2019).

Considering the varied roles of TET enzymes in biological processes, their emergence as critical therapeutic targets for illnesses such as cancer is not unexpected (X. Zhang et al. 2023). Modulating TET activity or targeting TET-influencing pathways is regarded as a possible therapeutic method for cancer treatment in the future (Kaplánek et al. 2023; López-Moyado et al. 2024; Salmerón-Bárcenas et al. 2023; X. Zhang et al. 2023). Compounds that directly or indirectly modify TET activity, together with certain inhibitors, have been demonstrated to influence many cancer hallmarks.

## Epi-nutrients upregulating epigenetic enzymes

Nutrition plays a pivotal role in gene regulation, as highlighted by various in vitro and in vivo investigations revealing that dietary substances can regulate the function of epigenetic enzymes such as HDACs and DNMTs (Casari et al. 2024; Khan et al. 2021; Pandey et al. 2012; Wu et al. 2023). Moreover, growing epidemiological evidence points to the connection between diet quality and epigenetic phenotypes linked to tumor prevention (Bordoni et al. 2024).

These enzymes modify DNA and histone structures, influencing gene activity while preserving the underlying DNA sequence (Reva et al. 2023). Specific nutrients have been identified as significant activators of these enzymes, establishing a link between dietary intake and gene expression modulation (Bordoni and Gabbianelli 2019). One prominent example is folate, which serves as a methyl donor essential for activating DNMTs, the enzymes responsible for DNA methylation (Mavioglu et al. 2022). It provides methyl groups that allow DNMTs to add methyl tags to DNA, a process that can lead to gene silencing or reduced gene expression (Cappuccilli et al. 2020; Lim et al. 2019). Other nutrients, including choline, zinc, and vitamin B12, also support DNMT activity, contributing to proper DNA methylation patterns that are critical for regulating gene expression (Allison et al. 2021). In addition to DNA methylation, dietary components can activate histone-modifying enzymes such as HATs (Shafabakhsh et al. 2019). HATs transfer acetyl groups to histone proteins, leading to chromatin relaxation and increased gene expression. Compounds such as resveratrol (found in grapes) and curcumin (found in turmeric) have demonstrated the ability to activate HATs, promoting histone acetylation and thereby influencing gene transcription (Qi et al. 2023; Shafabakhsh et al. 2019).

The activation of these epigenetic enzymes by specific nutrients highlights how diet can modulate gene expression and potentially reduce disease risk, such as cancer, cardiovascular diseases (CVDs), T2DM, neurodegenerative disorders, obesity, and metabolic syndrome (Roberti et al. 2021). This knowledge opens avenues for dietary interventions to influence gene regulation and mitigate disease risk (Zaiou et al. 2021). By understanding the activation pathways of epigenetic enzymes, researchers can develop targeted dietary approaches to support health and prevent disease (Cassotta et al. 2021).

It is interesting to note that innovative formulations are being developed to improve the bioavailability of bioactive compounds and the targeting of cancer cells by passive or active targeting to inhibit the development of cancer and tumorigenesis (Opara et al. 2025; Tuli et al. 2023). Poly(lactideco-glycolide) (PLGA) and mesoporous silica (MSN) nanoparticles can be modified by adding ligands to their surfaces that can bind to cancer cell receptors, such as ligands for folic acid and its receptors (FRα) overexpressed in breast cancer and glioblastoma cells (Ramalho et al. 2024; Tagde et al. 2020). Besides, bioactive compounds can also be entrapped in nanogels sensitive to acidic pH in the tumor microenvironment of prostate cancer cells, where they can be degraded (Liga and Paul 2025; Madhusudana Rao et al. 2015). Interestingly, emerging research indicates that cancer stem cells (CSCs) can also be targeted by bioactive compounds that can protect against cancer development (Opara et al. 2025). In fact, natural phytochemicals can both directly and indirectly impact non-coding RNAs and reduce cancer progression by regulating the expression of oncogenic and tumor-suppressor miRNAs (Tuli et al. 2023).

Moreover, the delivery of anti-miRNAs or miRNA mimics by nanoparticles or other delivery systems (graphene oxide) represents a therapeutic approach that allows epigenetic reprogramming of cancer cells and increases drug sensitivity. In acute myeloid leukemia (AML), gold nanoparticles (AuNPs) were modified by encapsulating anti-miR-221 and adding to their surfaces DNA aptamer AS1411, which can interact specifically with nucleolin in AML cells, thereby targeting the nucleolin/miR-221/NF-κB/DNMT1 signaling pathway responsible for leukemogenesis (Deng et al. 2018). In glioblastoma, miRNA mimics were used to restore the dysregulation of miRNAs (miRNA-7), thus improving the regulation of target genes'expression, like *PTEN* (Kutwin et al. 2024). Thus, innovative formulations have the potential ability to both prevent cancer recurrence and inhibit tumor progression while reducing side effects in healthy tissue.

### Epi-nutrients as activators of epigenetic enzymes

The role of specific nutrients as activators of epigenetic enzymes is a growing area of investigation that sheds light on the intricate relationship linking nutrition and gene regulation (Campit et al. 2020). Some nutrients can directly engange and turn epigenetic enzymes, which affect their activity and change epigenetic modifications (Abdul et al. 2017). For example, it has been demonstrated that sirtuins, a type of histone deacetylase involved in the control of gene expression, are activated by resveratrol, a polyphenol naturally present in grapes and red wine. Resveratrol may impact histone acetylation patterns and gene transcription by promoting sirtuins (B. Salehi et al. 2018). Similarly, genistein, an isoflavone found in soybeans, has been found to activate DNMTs (Venturelli et al. 2013). This activation can alter the methylation status of specific genes, leading to changes in gene expression (Venturelli et al. 2013).

Moreover, curcumin, a compound in turmeric, has been identified as a potential activator of histone-modifying enzymes, further underscoring the role of dietary components in epigenetic regulation (Link et al. 2010; vel Szic et al. 2015). These compounds are critical in regulating S-adenosylmethionine (SAM) availability, a primary methyl donor for DNA and protein methylation (Wu et al. 2006). Nutritional deficiencies during critical developmental periods can alter DNA methylation patterns, leading to increased disease susceptibility. For example, restrictive diets low in methionine and B vitamins during the periconceptual period in sheep have been associated with altered DNA methylation, hypertension, and insulin resistance in offspring (Sinclair et al. 2007).

Conversely, maternal diets rich in choline, betaine, vitamin B12, and folate enhance DNA methylation, reduce gene expression of obesity-related genes, and delay childhood obesity onset (Sinclair et al. 2007). Additionally, maternal supplementation with methyl donors can have transgenerational effects, with epigenetic changes passing to subsequent generations (Cropley et al. 2006).

SAM, generated from methionine by SAM synthase (methionine adenosyltransferase) (Wu 2010), is also essential for producing compounds like creatine, taurine, cysteine, and polyamines—substances critical for vascular health and cellular growth (Wu et al. 2006). In particular, polyamines are essential for vascular remodeling and endothelial cell proliferation (Li et al. 2002). It is important to note that the production of these bioactive substances is significantly impacted not only by the physiological and nutritional status of the organism but also by the amount of methyl donors available. The total amount of SAM available for DNA or protein methylation decreases with dietary deficiency of taurine or cysteine, as these nutrients are synthesized to a greater extent from methionine in vivo. The metabolism of one-carbon units can also be disrupted by insufficient nutrition and the production of glycine and serine (Wu et al. 2006). Consequently, deficiencies of amino acids may alter both histone modifications and DNA methylation, which in turn may alter epigenetic coding (Oommen et al. 2005).

#### Mechanisms of Epi-nutrient activators on epigenetic enzymes

Epigenetic mechanisms like DNA methylation and histone modifications act synergistically to control gene expression (Dorna et al. 2025). Typically found at CpG dinucleotides, DNA methylation can silence genes when found in promoter regions. Chromatin accessibility and structure are influenced by histone modifications such as phosphorylation, methylation, and acetylation (Ur Rehman et al. 2025). These processes interplay functionally rather than being mutually exclusive. Through chromatin condensation, for example, methylated DNA regions frequently attract HDACs, enhancing transcription inhibition (Davie et al. 2025; Schulz and Hoffmann 2025).

Epigenetic enzymes, notably HATs, HDACs, DNMTs, and HMTs, modify DNA and histone patterns to control gene expression (Mierziak et al. 2021). Nutrients can act as modulators or activators of these enzymes through complex mechanisms, either by directly enhancing the enzyme's catalytic activity or by indirectly improving enzyme function through metabolic or molecular modulation that alters chromatin dynamics and transcriptional regulation (Mahmoud and Ali 2019; Tiffon 2018).

Regarding biochemical mechanisms, one of the primary biochemical processes involves using metabolites derived from nutrients as cofactors or substrates for epigenetic enzymes. For instance, the one-carbon metabolic pathway produces SAM, which serves as a methyl donor necessary for methylation processes catalyzed by DNMT and HMT. Similarly, histone acetylation by HATs employs acetyl-CoA, generated from glycolysis and fatty acid oxidation, as a crucial substrate. The energy status of the cell influences the availability of NAD^+^, which, in turn, impacts the activity of sirtuins (class III HDACs) (Mahmoud and Ali 2019; Remely et al. 2015). Betaine, vitamin B12, and choline serve as examples of micronutrients that act as methyl donors and cofactors to facilitate DNA methylation. Nutrients can influence gene expression profiles and both cellular and physiological processes through DNA methylation (Haro et al. 2019; Mahmoud and Ali 2019; Mierziak et al. 2021; Seidel et al. 2012).

Nutrients can affect epigenetic enzymes by altering the transcription of their coding genes which relate to transcriptional mechanisms. The expression of epigenetic regulators such as HDAC1, DNMT1, and TET enzymes can be increased or decreased by specific bioactive dietary components. Signaling cascades linking transcription factors and nutritional sensing are often the mechanism by which this regulation is achieved (Mierziak et al. 2021; Ramazi et al. 2023; Wu et al. 2022). For example, eicosapentaenoic acid (EPA) and docosahexaenoic acid (DHA) are omega-3 fatty acids that can modulate transcription factor activities like NF-κB and peroxisome proliferator-activated receptors (PPARs), thereby impacting the expression of genes involved in inflammation, metabolism, and immune response (Adkins and Kelley 2010). the modulation of histone modifications. Histones represent a group of proteins that DNA wraps around to form chromatin. Different epigenetic changes, such as acetylation, methylation, and phosphorylation, can occur on histone proteins, altering the chromatin structure and accessibility of genes (Ramazi et al. 2020).

Therefore, changes in gene expression due to dietary modulation of epigenetic marks may contribute to diseases such as cancer, CVDs, and neurodegenerative disorders (Wu et al. 2022). Targeted dietary interventions to modulate epigenetic activity hold promise for therapeutic strategies and disease prevention (Chiacchiera et al. 2020). Furthermore, the implications of nutrient-induced epigenetic changes are far-reaching. Nutrient-modulated gene expression can impact cellular development, differentiation, metabolism, and disease susceptibility (Guilloteau et al. 2009). For example, sulforaphane and folate have been shown to impact epigenetic patterns by modulating histone acetylation and DNA methylation, respectively (Hsieh et al. 2025; Kumari et al. 2022).

#### Nutrient-mediated enzyme activation and its impact on gene expression and therapeutic applications

Understanding how specific nutrients activate epigenetic enzymes gives us valuable information about the diet's impact on gene regulation, emphasizing the potential of personalized nutrition for promoting health and preventing disease (Dauncey 2014; Grazioli et al. 2017). Indeed, several nutrients have been identified to possess activator properties, playing a critical role in epigenetic regulation by influencing gene expression and cellular function (Ayissi et al. 2014). Some notable examples include:

##### Folate

Folate is an essential methyl donor in the one-carbon metabolic pathway and therefore a crucial component of epigenetic regulation (Gurugubelli and Ballambattu, 2024; Liu and Ward 2010). DNMTs mediate DNA methylation, in which folate promotes the synthesis of SAM. Normal methylation patterns are maintained by adequate folate levels, but abnormal hypermethylation or global hypomethylation of TSGs promoters can result from folate deficiency (Joseph et al. 2018). These changes have been associated with inadequate gene regulation and elevated cancer risk. According to investigations, folate-mediated methylation affects genes such as *p16*, *hMLH1*, and *MGMT*, and can alter a patient's vulnerability to breast and colorectal cancers (Chen et al. 2012; Sanchez et al. 2017).

##### Vitamin B12

Vitamin B12 works synergistically with folate as a cofactor in converting homocysteine to methionine, which is essential for the synthesis of SAM, the primary methyl donor for DNA and histone methylation. Deficiency in vitamin B12 can disrupt these methylation processes, influencing gene regulation and potentially causing neurological disorders (Maddocks et al. 2016).

##### Choline

Choline, an essential nutrient for cell membrane structure and function, also serves as a methyl donor in DNA and histone methylation. Deficiency in choline has been associated with altered DNA methylation patterns, affecting gene expression and contributing to liver and neurological disorders (Mehedint and Zeisel 2013).

##### Resveratrol

This polyphenol activates sirtuin 1 (SIRT1), an NAD^+^-dependent deacetylase that influences histone acetylation and gene silencing (Li et al. 2013; Scuto et al. 2013). By activating SIRT1, resveratrol modulates gene expression associated with aging, inflammation, and metabolic health (Gao and Tollefsbol 2015; Mongioì et al. 2021; Venturelli et al. 2013; Zhou et al. 2021).

##### Curcumin

**Fig. 3:**
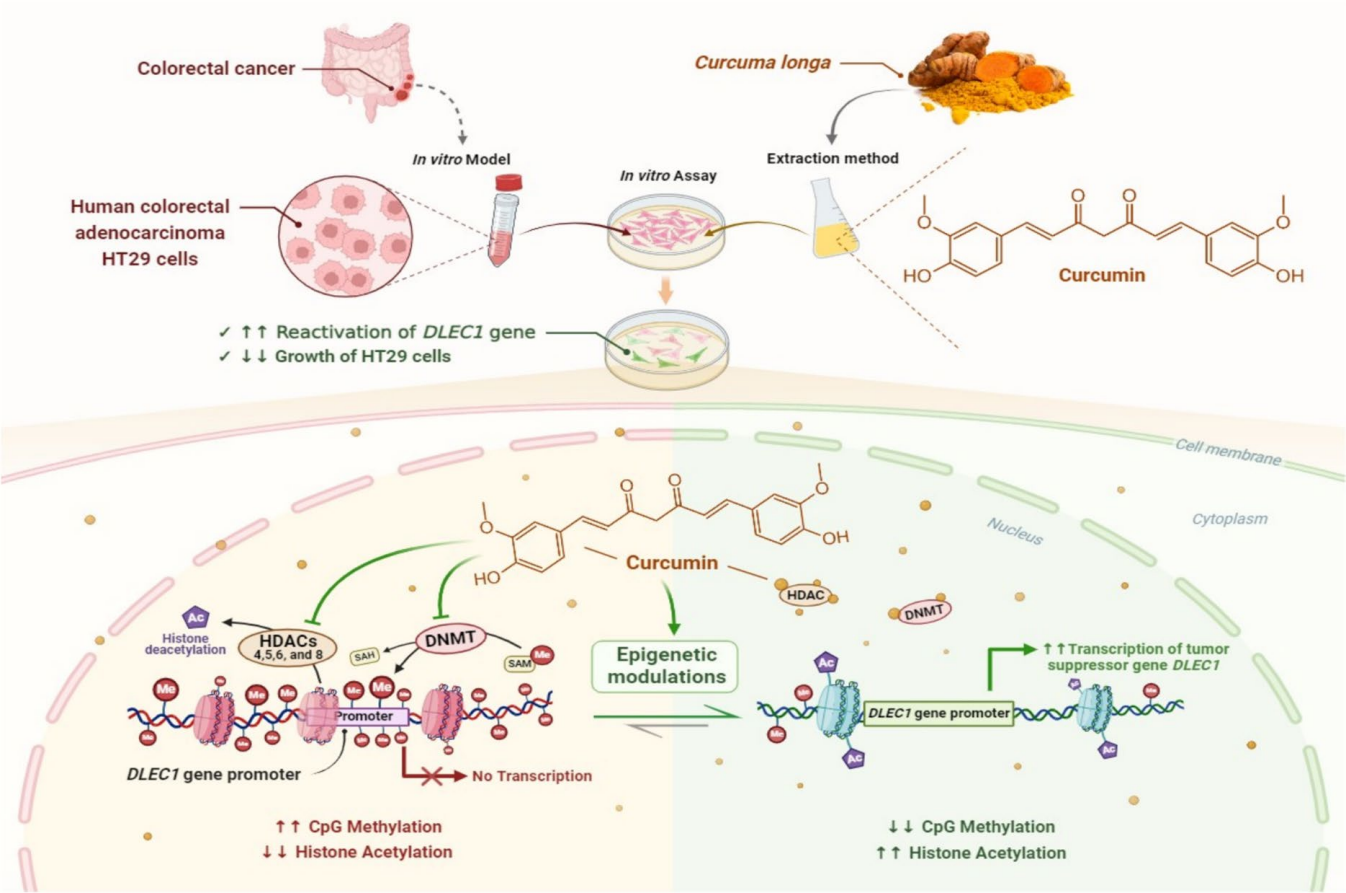
Schematic representation of the specific anticancer effect of curcumin on HT29 human colon cancer cells by focusing on its action on the *DLEC1* tumor suppressor gene through epigenetic reactivation. Using human colorectal adenocarcinoma HT 29 cells, the main ingredient of *Curcuma longa*, curcumin, has been demonstrated to be a powerful anticancer molecule that monitors numerous epigenetic modulations in vitro. The tumor suppressor gene *DLEC1* exhibits decreased transcriptional activity and promoter hypermethylation in a number of malignancies, such as colorectal cancer. In this sense, curcumin downregulated CpG methylation of the *DLEC1* promoter after a 5-day treatment period, which correlated with an upregulation of *DLEC1* mRNA expression in HT29 cells. In addition, curcumin decreased HDAC4, 5, 6, and 8 and DNMT expressions. ***Abbreviations:*** DNMT, DNA methyltransferases; HDAC, Histone deacetylase; DLEC1, deleted in lung and esophageal cancer 1; SAM, S-adenosylmethionine; SAH, S-adenosylhomocysteine

Curcumin, the active compound in turmeric, modulates HATs and HDACs, impacting gene expression linked to inflammation, cancer, and neurodegenerative diseases (Hassan et al. 2019) (Fig. 3).

##### Selenium

Selenium, a component of selenoproteins such as glutathione peroxidase, influences gene expression by reducing oxidative stress and promoting antioxidant defense, particularly relevant in inflammation-related gene regulation.

These nutrients significantly influence gene expression through the activation of epigenetic enzymes. Certain nutrients and bioactive compounds can alter substrate availability or directly modify enzymes involved in histone modifications and DNA methylation, ultimately impacting cellular function and health (Choi and Friso 2010; Tiffon 2018).

### *Epi-Nutrients as inhibitors of epigenetic enzymes*

Nutrients contribute significantly to health beyond providing basic sustenance; they interact at the cellular level to influence gene regulation, often through epigenetic pathways (Block et al. 2011). Nutrients can impact epigenetic regulation primarily by inhibiting key enzymes, such as HAT, HDAC, and DNMT, or by altering substrate availability necessary for these enzymes’ activities. By modulating gene expression, these nutrients affect overall health and longevity (Tiffon 2018).

The influence of dietary factors on disease susceptibility and phenotype has been widely studied, with compelling evidence supporting their long-term effects on disease predisposition and lifespan (Alzeer, 2025). Nutrients with methyl-donating potential, such as choline, also impact DNA methylation status and gene expression (Carlberg, 2023; Carlberg et al. 2020). Adequate intake of these nutrients is particularly essential during early pregnancy for fetal development, as they are crucial in establishing methylation patterns that affect health and disease susceptibility later in life. Notably, an in vivo study demonstrated that maternal deficiency in methyl-donating nutrients during the periconceptional period led to significant alterations in the DNA methylation and phenotype of offspring (Choi and Friso 2010).

Current investigations have deepened our understanding of the interplay between nutrients and epigenetic regulation, illustrating that certain nutrients not only activate but also inhibit epigenetic enzymes, thereby modulating gene expression (Baek and Kim 2017). Below are key examples of nutrients with inhibitory properties on epigenetic enzymes:

#### Epigallocatechin-3-gallate (EGCG)

**Fig. 4:**
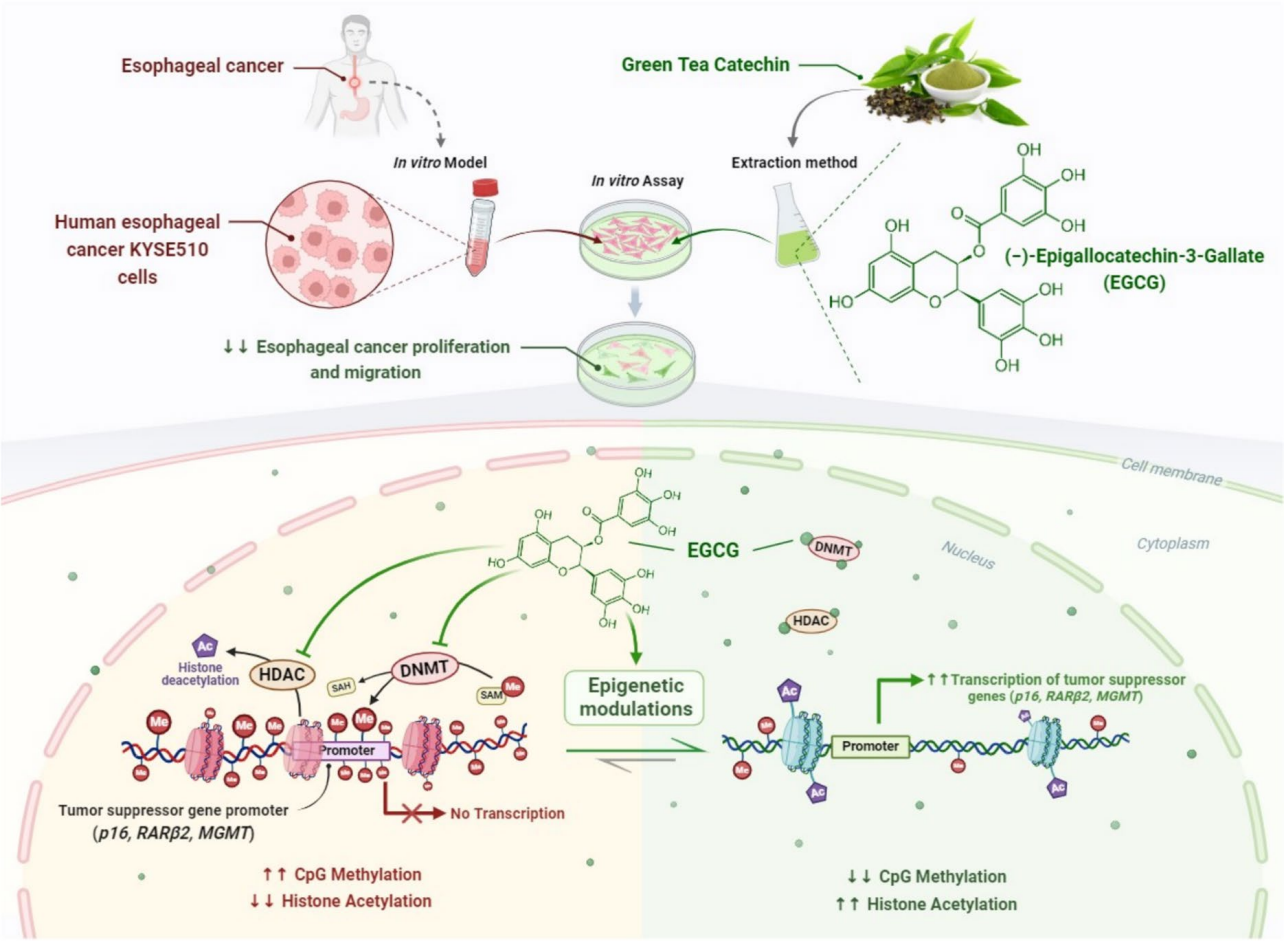
Schematic description of epigenetic modifications and induction of tumor suppressor genes (*p16*, *RARβ2*, *and MGMT*) by the catechin molecule in green tea EGCG. Hypermethylation of *MGMT*, *RARβ*, and *p16* genes was reversed in KYSE 510 human esophageal cancer cells exposed to 5–50 μM EGCG for 12–144 h in a concentration- and time-dependent manner. The phenomenon was associated with mRNA expression of these genes, as shown by RT-PCR. In KYSE 150 esophageal cancer cells, EGCG induces reactivation of certain genes that are silenced in terms of methylation. The findings indicate that EGCG could be used to prevent or reverse gene silencing in the fight against the onset of tumorigenesis, as it can suppress DNA methylation. ***Abbreviations:*** DNMT, DNA methyltransferases; HDAC, Histone deacetylase; DLEC1, deleted in lung and esophageal cancer 1; EGCG, (-)-epigallocatechin-3-gallate; RARβ, Retinoic acid receptor beta; MGMT, O(6)-methylguanine methyltransferase; SAM, S-adenosylmethionine; SAH, S-adenosylhomocysteine

EGCG, the principal polyphenol in green tea, has been shown to inhibit DNMTs, which are responsible for adding methyl groups to DNA (Fang et al. 2003) (Fig. 4). By suppressing DNMT activity, EGCG alters DNA methylation patterns, potentially modifying gene expression. This property has made EGCG a molecule of interest for therapeutic use as a natural epigenetic modulator, with potential applications in cancer prevention and treatment (Singh et al. 2011).

#### Quercetin

Quercetin, a flavonoid present in a variety of fruits, vegetables, and grains, acts as an inhibitor of both DNMTs and HDACs. By targeting these enzymes, quercetin modulates DNA methylation and histone acetylation, affecting gene expression related to inflammation, cancer, and cardiovascular health. Quercetin’s dual inhibitory effect on DNMTs and HDACs has generated interest in its potential as a nutraceutical in personalized nutrition and preventive medicine (Russo et al. 2017).

#### Genistein

Genistein, an isoflavone found in soy, is another nutrient with inhibitory effects on DNMTs and HDACs. This nutrient influences DNA methylation and histone acetylation, thereby affecting gene expression profiles relevant to cancer prevention and therapy. Genistein’s modulation of epigenetic mechanisms provides insight into its role as a preventive agent against cancer and its potential application in targeted nutrition strategies (Pudenz et al. 2014).

The identification of dietary components as inhibitors of epigenetic enzymes highlights the complicated relationship between nutrition and gene regulation. These findings offer promising avenues for targeted interventions and personalized approaches to health, especially in the context of disease prevention and management (Rinaldi et al. 2018). Dietary inhibitors of epigenetic enzymes offer new perspectives on leveraging nutrition as a complementary tool for modulating gene expression and improving health outcomes.

#### Mechanisms of Epi-nutrient inhibitors on epigenetic enzymes

Research into the role of nutrients in inhibiting epigenetic enzymes is rapidly evolving, with implications for gene regulation, disease prevention, and therapeutic applications (Choi and Friso 2010). Traditionally, nutrients have been known to support enzyme activity, yet emerging evidence reveals their potential as inhibitors of key epigenetic enzymes, offering new insights into gene expression modulation (Prachayasittikul et al. 2017). In this section, we discuss the pathways through which specific nutrients inhibit epigenetic enzymes such as DNMTs, HATs, and HDACs, thereby impacting gene expression (Tiffon 2018).

One prominent mechanism by which nutrients inhibit enzymes is by suppressing their expression levels. Certain dietary compounds, such as genistein, an isoflavone in soy, have been found to downregulate DNMT expression. This modulation of DNMT activity alters DNA methylation, which in turn impacts gene silencing or activation, ultimately influencing cellular function and potentially reducing cancer risk (Li & Tollefsbol 2010). Another mechanism involves nutrient competition at enzyme cofactor binding sites. For instance, polyphenols like quercetin and resveratrol act as competitive inhibitors of HMTs by occupying these binding sites, thereby reducing enzymatic activity and altering histone methylation patterns associated with disease phenotypes (Gerhäuser 2012; Li and Tollefsbol 2010; Patra et al. 2021).

Furthermore, nutrient intake affects the availability of substrates essential for enzyme function. By altering the levels of these substrates, nutrients can indirectly inhibit the enzymatic reactions (Kinnaird et al. 2016). For instance, nutrients involved in the synthesis of SAM, such as choline, are critical methyl donors in methylation reactions. Low levels of these nutrients can result in diminished SAM, reducing methylation activity and leading to broader changes in DNA and histone methylation, which are vital for healthy gene expression (Abbasi et al. 2018).

**Fig. 5:**
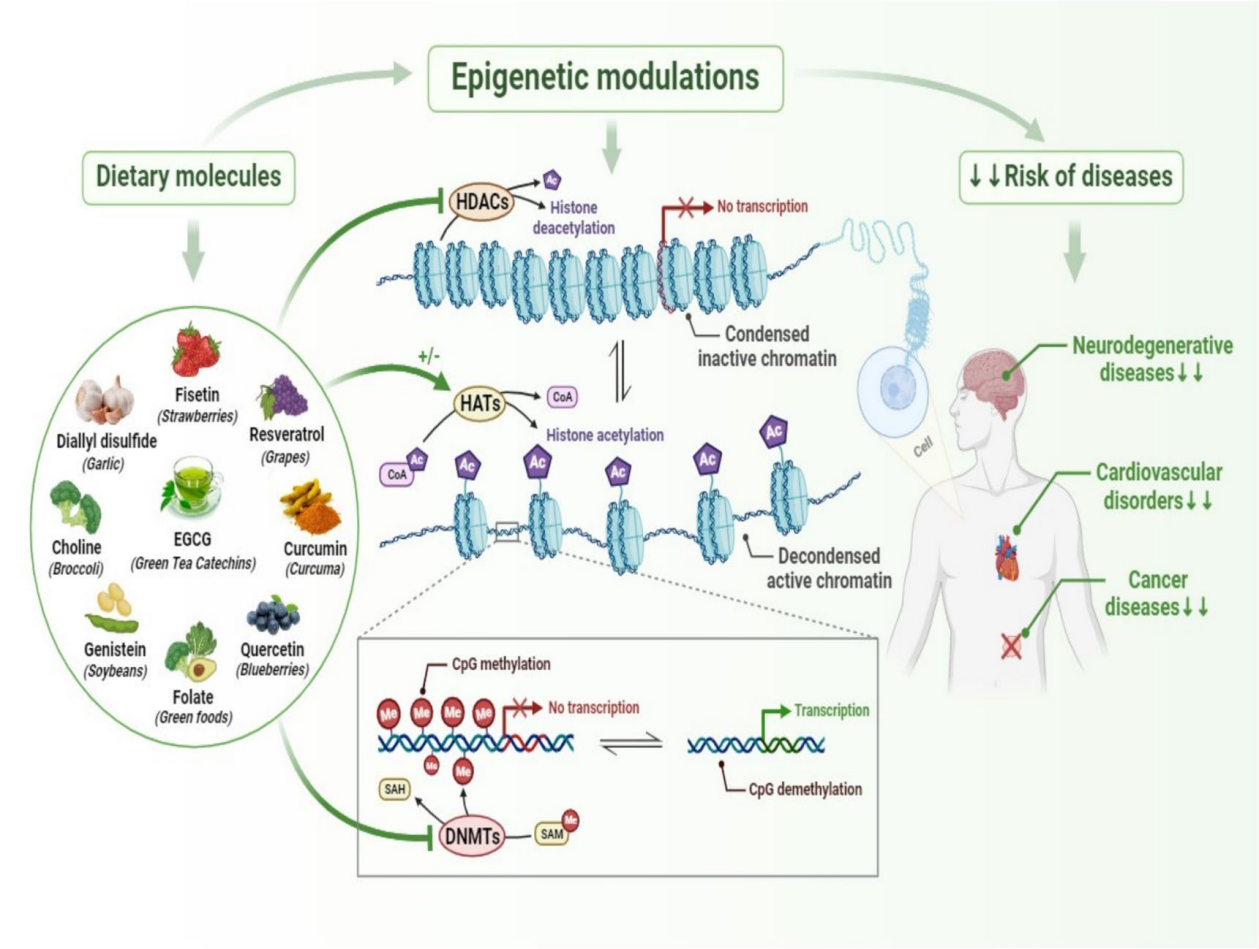
Proposed illustration of a range of phytochemicals originating from dietary sources that have a beneficial effect on the prevention of various diseases by effectively modulating epigenetic enzymes. Cancer, cardiovascular disease, and neurodegenerative impairment are among the diseases that have been linked to abnormal epigenetic changes. Therefore, by inhibiting epigenetic enzymes such as DNMT, HDAC, or HAT, bioactive molecules from dietary foods (such as quercetin, EGCG, genistein, quercetin, resveratrol, choline, and green tea catechins) can potentially have a direct therapeutic effect. ***Abbreviations:*** EGCG, epigallocatechin-3-gallate; DNMT, DNA methyltransferase; HDAC, histone deacetylase; HAT, histone acetyltransferase; SAM, S-adenosylmethionine; SAH, S-adenosylhomocysteine

The effects of nutrient inhibition on epigenetic enzymes have major implications for human health, with studies linking abnormal epigenetic modifications to diseases like cancer, CVD, and neurodegeneration (Joven et al. 2014). Catechins in green tea, for instance, show potential in modulating epigenetic patterns associated with cancer, demonstrating inhibitory activity against certain histone-modifying enzymes, which has sparked interest in their application as dietary epigenetic modifiers (Hwang et al. 2017) (Fig. 5).

#### Nutrient-mediated enzyme inhibition and its impact on gene expression and therapeutic applications

The inhibitory effects of nutrients on epigenetic enzymes have profound impacts on gene expression, creating possibilities for therapeutic applications (Lal et al. 2022). By modulating epigenetic modifications, nutrients influence how accessible genes are to transcriptional machinery, thus regulating gene expression patterns that are pivotal in development and disease (Bhat and Kapila 2017) (Fig. 5).

One primary outcome of inhibiting DNMTs, for example, is the hypomethylation of DNA. Normally, DNA methylation silences gene expression (Li and Tollefsbol 2010). However, when DNMT activity is suppressed—such as by resveratrol—DNA undergoes hypomethylation, potentially reactivating TSGs and contributing to cancer prevention (vel Szic et al. 2015). Such modifications demonstrate the therapeutic value of nutrient-based DNMT inhibitors in reestablishing healthy gene expression patterns disrupted in diseases like cancer.

In addition to DNA methylation, HDAC inhibition by nutrients can also influence gene expression. HDACs typically compact chromatin by removing acetyl groups from histone tails, reducing gene accessibility to transcription factors (Biel et al. 2005). Nutrients, like curcumin, have been shown to inhibit HDACs, promoting histone acetylation and activating transcription of genes involved in immune response, apoptosis, and cell cycle regulation (Biel et al. 2005). This effect is valuable for therapeutic strategies in cancer and inflammatory diseases, where HDAC inhibition can restore normal cellular functions (Perri et al. 2017).

The potential therapeutic applications of nutrient-mediated inhibition of epigenetic enzymes are wide-ranging. Dysregulation of epigenetic processes is implicated in several pathologies, including CVDs, tumors, neurodegenerative disorders, and metabolic disorders. Targeting these processes with nutrients offers a promising avenue for therapeutic interventions (Bhat and Kapila 2017). Indeed, these nutrient-mediated effects on epigenetic enzymes hold therapeutic promise for numerous conditions. In cancer, the reactivation of TSGs through nutrient-driven DNMT and HDAC inhibition can help restore cellular homeostasis and potentially reduce tumor growth (Perri et al. 2017). Similarly, in neurodegenerative diseases, aberrant epigenetic modifications contribute to disease progression, and nutrient inhibitors could offer neuroprotection by modulating genes involved in neuronal health and function (Meadows 2021).

In metabolic disorders such as diabetes and obesity, nutrients that target epigenetic enzymes may help maintain metabolic balance. In fact, bioactive compounds in foods that inhibit HDACs and DNMTs could modulate genes controlling glucose and lipid metabolism, improving disease outcomes (Li et al. 2022). As such, nutrient-driven epigenetic modulation represents a promising avenue for therapeutic interventions that could complement conventional treatments, promoting health and potentially reversing disease-related epigenetic marks.

In summary, understanding how nutrients interact with epigenetic enzymes offers exciting opportunities for personalized nutrition and disease prevention. By targeting specific enzymes, nutrients can modulate gene expression to promote health, mitigate disease risk, and contribute to longer-term therapeutic outcomes (Lal et al. 2022).

### Epi-nutrients as epigenetic enzyme substrates

Nutrients play versatile roles in epigenetic regulation, with certain nutrients directly serving as substrates for key enzymes involved in epigenetic modifications, highlighting an intricate interplay between diet, metabolism, and gene regulation (Reid et al. 2017). These nutrients participate in fundamental biochemical pathways and contribute to the dynamic regulation of gene expression.

One prominent example is acetyl-CoA, a critical metabolite in cellular energy production and metabolic pathways (Moffett et al. 2020). Acetyl-CoA acts as a substrate for HATs, which are used to transfer acetyl groups to histone proteins. This process of histone acetylation relaxes chromatin structure, making DNA more accessible for transcription and thereby promoting gene expression (Luebben et al. 2010). Consequently, the availability of acetyl-CoA can significantly influence histone acetylation and gene activation, linking cellular energy status to gene regulation (Lei et al. 2021).

As previously mentioned, SAM, a derivative of methionine, is an essential methyl donor that serves as a substrate for DNMTs (Bekdash 2023). DNMTs transfer methyl groups from SAM to specific cytosine residues in DNA, resulting in DNA methylation (Bekdash 2023). The presence and synthesis of SAM highlight the nutritional dependency of DNA methylation and suggest that methionine intake can directly impact DNA methylation patterns.

Certain vitamins also serve as substrates in these epigenetic reactions (Meier 2013). Folate, a B vitamin participates in one-carbon metabolism and supplies methyl groups essential for DNA methylation. In its active form, 5-methyltetrahydrofolate, it transfers a methyl group to homocysteine to form methionine, which is then converted to SAM (Steluti et al. 2020). This pathway emphasizes the role of this vitamin in maintaining adequate methyl donor levels for DNA methylation, underscoring its importance in epigenetic regulation.

Alpha-ketoglutarate (α-KG), a key metabolite in the tricarboxylic acid (TCA) cycle, serves as a substrate for TET enzymes, which are responsible for DNA demethylation (Chia et al. 2011). TET enzymes require α-KG for their catalytic function, enabling the removal of methyl groups from DNA and facilitating dynamic changes in DNA methylation (Chia et al. 2011). This relationship between α-KG and DNA demethylation underlines the direct influence of metabolic intermediates on epigenetic modifications.

The involvement of these substrates in epigenetic processes illustrates the intricate link between nutrition, cellular metabolism, and gene regulation (Anderson et al. 2012). Nutrients not only fuel enzymatic reactions but also participate directly in epigenetic modifications, influencing gene expression patterns and cellular behavior (Campit et al. 2020). Proper nutrient availability is thus essential for maintaining balanced epigenetic modifications that contribute to overall health.

### Epi-nutrients as epigenetic enzyme cofactors

In addition to functioning as substrates, nutrients also serve as vital cofactors that assist various enzymes in catalyzing epigenetic processes, further illustrating the complex role of nutrition in regulating gene expression (Donohoe and Bultman 2012). Cofactors are essential molecules that aid enzymes in achieving optimal catalytic activity, facilitating specific biochemical reactions in the body. Within the realm of epigenetics, certain vitamins and minerals act as cofactors for enzymes involved in DNA and histone modifications (Donohoe and Bultman 2012).

Vitamins B12 and folate exemplify this function, as they act as cofactors in the synthesis of SAM (Fiorito et al. 2014). Vitamin B12 is specifically required by methionine synthase to convert homocysteine to methionine, subsequently generating SAM (Guéant et al. 2013). Maintaining adequate levels of these vitamins is critical to the proper function of DNMTs and the maintenance of DNA methylation patterns (Guéant et al. 2013). Deficiencies in these cofactors can disrupt DNA methylation and potentially lead to aberrant gene expression patterns.

In addition to influencing DNA methylation, certain minerals act as cofactors for histone-modifying enzymes. Iron and α-KG, for example, serve as cofactors for TET enzymes involved in DNA demethylation, necessary for the oxidation steps that lead to the removal of methyl groups from DNA (Liu et al. 2022). This demethylation process enables more flexible regulation of gene expression and demonstrates how mineral availability directly affects gene accessibility and transcription (Liu et al. 2022).

Furthermore, zinc is a cofactor for HDACs, which remove acetyl groups from histone proteins, resulting in condensed chromatin and gene silencing. Adequate zinc levels are necessary for HDAC activity, and imbalances can lead to dysregulated chromatin structure and altered gene expression (Marín-García and Llobat 2021). The relationship between minerals like zinc and HDAC activity highlights the importance of micronutrients in maintaining controlled gene silencing.

This multifaceted role of nutrients as cofactors underscores their critical impact on gene regulation through epigenetic pathways. Imbalances in these nutrients, due to dietary deficiencies or metabolic issues, can lead to disruptions in epigenetic modifications, potentially contributing to various diseases (Anderson et al. 2012). Maintaining proper nutrient levels, therefore, not only supports metabolic health but also preserves the integrity of epigenetic processes, aiding in disease prevention and promoting cellular homeostasis.

These insights point to the potential of epi-nutrients to induce epigenetic enzyme modulation and provide a molecular basis for promoting treatment outcomes. The result is a growing evidence base demonstrating how these compounds can improve therapeutic outcomes in combination with standard cancer therapies.

## Synergistic involvement of Epi-nutrients with anti-cancer drugs

The potential benefits of combining phytonutrients with existing cancer drugs have been highlighted by recent preclinical and clinical investigations. By modulating critical molecular targets, restoring drug resistance, and reducing systemic toxicity, these bioactive ingredients offer not only their intrinsic epigenetic potential but also increase the efficiency of available chemotherapeutic drugs in direct or indirect ways (Roy et al. 2025). For example, in a preclinical study, curcumin was found to reduce levels of heat shock protein 27 (HSP27) and P-glycoprotein (P-gp), causing an increase in the sensitivity of colorectal cancer cells to the chemotherapeutic drug 5-fluorouracil (5-FU), which led to enhanced cell death in 5-FU-resistant HCT-8 cancer cells (He et al. 2020). Another research study has revealed that curcumin increases the anticancer activity of 5-FU by blocking the expression of the MDR1 gene. Curcumin suppresses cell proliferation and induces apoptosis in 5-FU-resistant colorectal cancer cell lines by increasing miRNAs that repress EMT and decreasing EMT expression (Salehi et al. 2018). Furthermore, resveratrol has been shown to reverse resistance to the chemotherapeutic drug doxorubicin in breast cancer by modulating the SIRT1/β-catenin signaling pathway, both in vitro and in vivo models (Jin and Liu 2018). Moreover, a recent multi-omics investigation demonstrated that EGCG enhances the epigenetic effect of decitabine in the treatment of acute myeloid leukemia by promoting the reactivation of TSGs (Addissouky, 2025). By modulating histone acetylation and DNA methylation, EGCG improves the effects of oxaliplatin and irinotecan and improves the response to chemotherapy (Zhao et al. 2022). Research has shown that tea, ginseng, and quercetin extracts improve the efficacy of chemotherapeutic drugs while influencing epigenetic regulation in the treatment of colorectal cancer. Ginsenosides from ginseng enhance the efficacy of cisplatin and 5-FU by affecting histone modifications and DNA methylation, thereby inhibiting tumor growth in colorectal cancer cell lines (HCT116, SW480, HT29) (Zhao et al. 2022). By controlling microRNA expression and chromatin remodeling in mouse xenograft models, quercetin has demonstrated synergy with doxorubicin, paclitaxel, and etoposide, reducing treatment resistance. This bioactive compound modulates numerous signaling pathways, including PI3K/AKT, NF-κB, P53, Wnt/β-catenin, MAPK, JAK/STAT, and the Hedgehog pathway, to exert its anticancer effects. Quercetin disrupts various intracellular signaling molecules, including VEGF, TNF-α, Bax, Bcl-2, and caspases (Asgharian et al. 2022).

In addition, the synergistic effect of adding all-*trans*-retinoic acid (ATRA), a bioactive vitamin A metabolite, to decitabine has shown promise for treating myelodysplastic syndromes and acute myeloid leukemia. Mechanistically, this combination induces a suppression of Nrf2 activation through activation of the RARα-Nrf2 complex, leading to accumulation of ROS (Wang et al. 2023). In the study by Čižauskaitė and his team, highly metastatic human colon carcinoma cells were treated with sulforaphane, a bioactive compound found in cruciferous vegetables, in combination with the FOLFOX regimen (a combination of 5-FU, leucovorin, and oxaliplatin) to demonstrate an additive anticancer effect. When comparing the combined treatment with FOLFOX alone, the combined compounds significantly reduced cancer cell migration and proliferation. According to these results, sulforaphane may enhance the therapeutic efficacy of conventional chemotherapy via additional mechanisms, such as apoptosis, oxidative stress, and possibly epigenetic regulation. This potentially offers an effective adjunctive approach to the treatment of colorectal cancer (Čižauskaitė et al. 2022).

## Epi-nutrients as epigenetic modulators in cancer diseases

Nutrition plays a fundamental role not only in gene expression but also in influencing individual susceptibility to diseases, including cancer. Specific dietary components, often termed epi-nutrients, have shown the potential to modulate epigenetic mechanisms, such as DNA methylation, histone modifications, and miRNA expression, that are critical in cancer prevention and progression (Andreescu et al. 2018; Bakrim et al. 2023; Paluszczak et al. 2010). Table 1 provides an overview of selected dietary compounds with recognized epigenetic effects on gene expression regulation in cancer contexts.

**Table 1:** Natural Bioactive Molecules and Their Roles in Epigenetic Modifications of Cancer-Associated Genes

Dietary bioactive compounds	Cancer cell lines	Models	Genes	Epigenetic modifications	References
Catechol-containing polyphenols Rosmarinic and ellagic acids	- Human breast cancer MCF7 cell line	MTT test	*RASSF1A* *GSTP1* *HIN1*	- Inhibited DNMT - Did not affect methylation profile or *GSTP1*, *HIN1*, or *RASSF1A* expression - Did not impact histone H3 global methylation	(Paluszczak et al. 2010)
Green tea catechin: (−)-Epigallocatechin-3-gallate (EGCG)	- Esophageal cancer KYSE 510 cells	RT-PCR DNMT activity assay	*p16 (CDKN2A)* *RARβ2* *MGMT*	- Suppressed DNMT - Reactivated TSGs expression (*p16 (CDKN2A)*, *RARβ2*, and *MGMT*)	(Fang et al. 2003)
	- Human epidermoid carcinoma A431 cells	RT-PCR Western blotting	*p16*^*INK4a*^ *Cip1*/*p21*	- Inhibited DNMT1, DNMT3a, and DNMT3b - Suppressed HDAC activity - Restored *p16*^*INK4a*^ and *Cip1/p21* expression	(Nandakumar et al. 2011)
	- Human breast cancer MDA-MB-231 and MCF7 cell lines	RT-PCR DNMT activity assay	*SCUBE2*	- Decreased DNMT activity and expression - Enhanced *SCUBE2* expression	(Sheng et al. 2019)
	- Oral squamous cell carcinoma cell lines - Human cervical cancer cells HeLa	MSP qRT-PCR RT-PCR	*RECK*	- Restored *RECK* gene's hypermethylation state - Markedly increased *RECK* mRNA levels	(Kato et al. 2008)
	- Non-small-cell lung cancer (NSCLC) cell lines H460 and A549	RT-PCR MSP	*WIF-1*	- Reactivated methylation of *WIF-1* promoter - Restored *WIF-1* expression	(Gao et al. 2009)
	- Human cervical cancer cells HeLa	RT-PCR MSPR	*RARβ* *CDH1* *DAPK1*	- Markedly reduced DNMT3B in a time-dependent manner - Inhibited HDAC1 activity - Reversed *RARβ, CDH1,* and *DAPK1* expression	(Khan et al. 2015)
Flavonoid: Quercetin	- Human colon cancer cell line RKO	MTT assay	*p16*^*INK4a*^	- Inhibited hypermethylation of *p16*^*INK4a*^ - Restored *p16*^*INK4a*^ expression	(Tan et al. 2009)
	- Human xenograft acute myeloid leukemia (AML)	Western blotting ChIP assay MSP	*DAPK1* *BCL2L11* *BAX* *APAF1* *BNIP3* *BNIP3L*	- Inhibited DNMT1 and DNMT3a expression - Caused H3 and H4 acetylation - Decreased DNMT/HDAC in vivo - Provoked demethylation of *BCL2L11*/*DAPK1* genes	(Alvarez et al. 2018)
	- Human cervical cancer cells HeLa	qRT-PCR DNMT activity assay	*GSTP1,* *SOCS1,* *MLH1,* *APC, CDH1, DAPK1,* *VHL,* *FHIT, PTEN, RASSF1, TIMP3*, *RARB, CDH13,* and *MGMT*	- Inhibited DNMTs, HDACs, and HMTs - Reduced DNA methylation globally - Decreased promoter methylation of TSGs	(Kedhari Sundaram et al. 2019)
	- Human breast cancer MDA-MB-231 cell line	qRT-PCR Western blotting	*p51* *p21* *GADD45* *FOXO3a*	- Enhanced *p53, p21,* and *GADD45* expression and activities - Activated FOXO3a function	(Nguyen et al. 2017)
	- Human lung epithelial BEAS-2B cells	RT-PCR Western blotting	*PDCD4*	- Suppressed transformation of malignant cells induced by Cr(VI) - Regulated miR-21-PDCD4 signaling pathway	(Pratheeshkumar et al. 2017)
Isothiocyanate: Sulforaphane	- LnCap prostate cancer cells	RT-PCR Western blotting HDAC activity assay	*p21*	- Inhibited HDAC and DNMT - Facilitated reexpression of silenced tumor suppressors	(Ho et al. 2011)
	- PC3, LnCap, and benign prostatic hyperplasia (BPH-1) cell lines	qRT-PCR Western blot MSP Global Methylation Status	*CCND2*	- Downregulated DNMT1, DNMT3a, and DNMT3b - Reduced methylation in regions of the *CCND2* promoter containing the c-Myc binding site and multiple binding sites Sp1	(Hsu et al. 2011)
	- Human breast cancer MDA-MB-231 and MCF7 cell lines	ChIP analysis Telomerase activity assay RT-PCR qRT-PCR	*hTERT*	- Decreased levels of DNMT1 and DNMT3a - Repressed *hTERT* - Increased levels of acetyl-H3, acetyl-H3K9, and acetyl-H4	(Meeran et al. 2010)
	- Prostate cancer TRAMP-C1 cells	MeDIP RT-PCR ChIP assay	*Nrf2* *NQO1*	- Restored *Nrf2* expression - Inhibited DNMT1 and DNMT3a - Inhibited CpGs in the promoter of *Nrf2*	(Zhang et al. 2013)
	- Human cervical cancer cells, HeLa	RT-PCR MSP	*RARβ* *CDH1* *DAPK1* *GSTP1*	- Inhibited DNMT3B expression and activity - Inhibited HDAC1 expression and activity - Enhanced and reactivated expression of *RARβ*, *CDH1, DAPK1*, and* GSTP1* - Induced methylation of genes studied	(Ali Khan et al. 2015)
	- Human breast cancer MDA-MB-231 and MCF7 cell lines	qRT-PCR MSP Western blotting	*CAV1*	- Exerted demethylation of *CAV1* promoter	(Deb et al. 2014)
Isoflavone: Genistein	- Esophageal squamous cell carcinoma cells (KYSE 51) - Prostate cancer LNCaP and PC3 cells	qRT-PCR MSP	*p16*^*INK4a*^ *RARβ2* *MGMT*	- Hypomethylated and reactivated silenced genes - Suppressed DNMT activity	(Fang et al. 2005)
	- Human prostate cancer cell lines	qRT-PCR ChIP assay	*BTG3*	- Decreased promoter methylation and reactivated *BTG3* expression - Enhanced acetylation levels of H3 and H4	(Majid et al. 2010)
	- Human prostate cancer cell lines	MSP Immunohistochemistry	*GSTP1* *RASSF1A* *EPHB2* *BRCA1*	- Demethylated *GSTP1* and *EPHB2* - Reactivated *GSTP1*/*EPHB2* expression	(Vardi et al. 2010)
	- Intraductal breast specimens	Methylation assessment	*p16* *RASSF1A* *RARβ2* *ER* *CCND2*	- Enhanced hypermethylation of *RARβ2* - Enhanced hypermethylation of *CCND2* - Caused specific dose-dependent alterations in the methylation of *RARβ2* and *CCND2* genes - Exhibited antiestrogenic activity	(Qin et al. 2009)
Polyphenol: Curcumin	- Breast cancer cell lines (HCC-38, UACC-3199, and T47D)	MSP qRT-PCR	*BRCA1* *TET1*	- Reactivated *BRCA1* expression - Decreased methylation of the DNA promoter in HCC 38 and UACC 3199 cells - Upregulated *TET1* expression - Promoted hypermethylation of *SNCG*	(Al-Yousef et al. 2020)
	- HeLa, MCF7, A549,and A431 human cancer cell lines	MSP qRT-PCR	*p21* (*WAF1*/*Cip1*) *KLF4*	- Enhanced p21 expression - Increased KLF4 expression - Increased the *p21* promoter - Demethylated promoter CGI	(Chatterjee et al. 2019a)
	- Human medulloblastoma cell lines DAOY, D283 Med, and D341 Med	HDAC activity assay	*PARP*	- Blocked HDAC4 expression - Enhanced tubulin acetylation - Increased PARP expression	(Lee et al. 2011)
	- Osteosarcoma cell lines U2OS and SaOS2 - Human breast cancer MCF7 cell line	p53 acetylation marks RT-qPCR Western blotting	*p53*	- Enhanced acetylated H3K18 and H4K16 - Inhibited cell growth - Altered expression and acetylation of p53	(Collins et al. 2013)
	- Breast carcinoma cell lines (MDA-MB-231, MCF-10A, or MCF-7)	RT-PCR	*RASSF1A*	- Inhibited DNMT - Restored RASSF1A expression	(Du et al. 2012)
	- Human colorectal adenocarcinoma HT29 cells	MSP methylated DNA immunoprecipitation	*DLEC1*	- Reduced CpG methylation of *DLEC1* - Inhibited DMNT - Suppressed HDAC4, 5, 6, and 8 - Restored *DLEC1* expression	(Guo et al. 2015)
	- Dalton’s lymphoma ascites cells	RT-PCR Western blotting	*p53*	- Enhanced p53 expression and activity	(Das and Vinayak 2015)
	- Lung cancer A549 and H460 cells	RT-PCR MSP	*RARβ*	- Decreased *DNMT3b* mRNA levels - Increased *RARβ* mRNA levels - Restored *RARβ expression*	(Jiang et al. 2015)
	- MDA-MB-231 cells	qRT-PCR ChIP assay	*DLC1* *EZH2*	- Restored *DLC1* expression by suppressing EZH2	(Zhou et al. 2020)
	- Prostate cancer TRAMP-C1 cells	MeDIP qRT-PCR ChIP assay	*Nrf2* *NQO1*	- Inhibited DNMT - Reversed hypermethylation of CpG island of *Nrf2* gene - Induced Nrf2/NQO1 expression	(Khor et al. 2011)
	- Cervical cancer cell line SiHa	HDAC activity assay	*HPV E6/E7* *PRb* *p21* *p27 CCND1 CDK4 MRP1 Pgp1*	- Inhibited HDAC1 and HDAC2 activity - Inhibited HPV E6/E7 oncoprotein expression - Reduced MRP1/Pgp1 expressions	(Roy and Mukherjee 2014)
Stilbenes: Resveratrol	- Prostate cancer cell lines	RT-PCR ChIP assay	*MTA1*	- Decreased MTA1 protein expression - Suppressed MTA1/NuRD - Inhibited HDAC activity - Restored p53 - Destabilized HDAC1 levels	(Kai et al. 2010, p. 53)
	- Human prostate cancer cells, DU145 and PC3M	MTA1 silencing Western blot	*MTA1* *PTEN*	- Restored acetylation and reactivation of PTEN - Inhibited MTA1/HDAC	(Dhar et al. 2015)
	- Acute lymphoblastic leukemia cell line (CCRF-CEM)	qRT-PCR MSP	*MDR1*	- Decreased *MDR1* expression - Unchanged methylation pattern	(Zadi Heydarabad et al. 2018)
	- Human breast cancer cell lines: MDA-MB468, MDA-MB231, and MCF7 - Human melanoma A2058	DNA methylation assay RT-PCR ChIP assay	*STAT3* *ERα*	- Mediated demethylation of TSGs - Mediated reactivation of TSGs - Increased CpG island methylation of TSGs - Regulated expression of TSGs and interacted with DNMT1	(Lee et al. 2012)
	- MDA-MB-231 and MCF-7 human breast cancer cells	RT-PCR ChIP assay	*BRCA1* *p53* *p21*^*CIP1*^	- Enhanced BRCA1, p53, and p21^CIP1^ expression - Reduced PRMT5 expression - Increased EZH2 expression	(Chatterjee et al. 2019b)
	- MCF-7 breast cancer cells	qRT-PCR ChIP assay Western blotting	*BRCA1*	- Inhibited DNMT1 - Antagonized *BRCA1* histone sequence alterations induced by TCDD	(Papoutsis et al. 2010)

**A**bbreviations: qRT-PCR: Quantitative real-time PCR; hTERT: Human telomerase reverse transcriptase; MSP: Methylation-specific PCR; ChIP: Chromatin immunoprecipitation; BPH-1: Benign prostatic hyperplasia epithelial cell line; BTG3: B-cell translocation gene 3; MTT: 3-(4,5-Dimethylthiazol-2-yl)−2,5-diphenyltetrazolium bromide; RASSF1A: Ras association domain family member 1; GSTP1: Glutathione S-transferase pi 1; Nrf2: Nuclear factor erythroid-2-related factor 2; RARβ2: Retinoid acid receptor β2; HIN-1: High in normal-1; DNMT: DNA methyltransferase; HDAC: Histone deacetylase; MGMT: O6-methylguanine-DNA methyltransferase; EPHB2: Ephrin B2; BRCA1: Breast cancer 1; ER: Estrogen receptor; CCND2: Cyclin D2; SNCG: Synuclein γ; KLF4: Kruppel-like factor 4; PARP: Poly (ADP-ribose) polymerase; MTA1: Metastasis-associated protein 1; NuRD: Nucleosome remodeling and deacetylation; MDR1: Multidrug resistance gene 1; BCL2L11: BCL-2-interacting mediator of cell death (BIM); DAPK1: Death-associated protein kinase 1; BNIP3: BCL2 interacting protein 3; APAF1: Apoptotic protease activating factor 1; BAX: BCL2 associated X, apoptosis regulator; DLEC1: Deleted in lung and esophageal cancer 1; WIF-1: Wnt inhibitory factor-1; SCUBE2: Signal peptide, CUB domain and EGF like domain containing 2; CDH1: Cadherin 1; HMTs: Histone methyltransferases; TSGs: Tumor suppressor genes; SOCS1: Suppressor of cytokine signaling 1; TIMP3: Tissue inhibitor of metalloproteinase 3; VHL: Von Hippel-Lindau tumor suppressor; APC: Adenomatous polyposis coli; MLH1: MutL Homolog 1; FHIT: Fragile histidine triad diadenosine triphosphatase; GADD45A: Growth arrest and DNA damage-inducible alpha; PDCD4: Programmed cell death 4; MeDIP: Methylated DNA immunoprecipitation; EZH2: Enhancer of zeste homolog 2; PRMT5: Protein arginine methyltransferase 5; TCDD: 2,3,7,8-Tetrachlorodibenzo-p-dioxin; CAV1: Caveolin-1; HPV: Human papillomavirus; E6/E7: Viral oncoproteins; PRb: Retinoblastoma protein; CDKN2A: Cyclin dependent kinase inhibitor 2 A; NQO1: NAD(P)H quinone dehydrogenase 1; FOXO3a: Forkhead box O3a; STAT3: Signal transducer and activator of transcription 3; DLC1: Deleted in liver cancer 1; CDK4: Cyclin-dependent kinase 4; MRP1: Multidrug resistance-associated protein 1; Pgp1: P-glycoprotein 1.

The emerging field of epinutrition explores how natural bioactives found in food can influence the epigenome, offering promising avenues for preventive nutrition. These compounds can intervene in various epigenetic pathways to modify gene expression in ways that may inhibit tumorigenesis.

### Natural bioactive compounds as modulators of DNA methylation in cancer

DNA methylation relies on methyl donors and cofactors to regulate DNMT activity effectively, supporting genomic stability and cellular differentiation (Zhang 2015). Dietary factors are critical in maintaining methylation balance (Andreescu et al. 2018; Bishop and Ferguson 2015), with SAM serving as the exclusive physiological donor of methyl groups for cytosine methylation (Feil and Fraga 2012). SAM synthesis from homocysteine requires methionine and various dietary precursors, such as choline and betaine (Zeisel 2009), with deficiencies impacting DNA methylation dynamics (Andreescu et al. 2018; Niculescu and Lupu 2011). Nutrients affecting DNA methylation are often categorized into three main groups: (i) methyl donors, which directly supply methyl groups for DNA methylation; (ii) modifiers of enzymes influencing methyl group availability; and (iii) DNMT inhibitors, which reduce methylation activity (Ho et al. 2011).

#### One-carbon metabolism nutrients

One-carbon metabolism nutrients, including folate, riboflavin, methionine, cobalamin (vitamin B12), and pyridoxine (vitamin B6), are pivotal for DNA methylation processes and have demonstrated cancer-preventive properties (Duthie 2011b; Mazzio and Soliman 2014). Epidemiological evidence suggests a protective effect of adequate folate levels against various cancers, notably breast and cervical cancers in premenopausal women, due to its role in maintaining proper methylation (Andreescu et al. 2018; Lillycrop and Burdge 2012; Maruti et al. 2009; Pembrey et al. 2014; Teegarden et al. 2012). Choline also contributes to the methyl pool as a precursor of methionine, while vitamin B12 and vitamin B6 are essential cofactors for methylation enzymes, ensuring efficient methylation cycles (Zhang et al. 2003). Riboflavin assists methylenetetrahydrofolate reductase (MTHFR) in synthesizing methionine, with riboflavin deficiencies shown to impede DNA methylation by restricting MTHFR activity (Stefanska et al. 2012).

#### Phytoestrogens

Phytoestrogens, like genistein and resveratrol, modulate estrogen-responsive genes by binding to estrogen receptors, influencing methylation patterns in cancer pathways (Mandal and Davie 2010). Genistein, a notable inhibitor of DNMTs, has been shown to block epigenetic mechanisms promoting cancer progression in prostate and esophageal cancer models, affecting promoter methylation and DNMT activity (Fang et al. 2005; Ho et al. 2011). Resveratrol, similarly, downregulates DNMT3b expression, particularly in tumor cells, thereby mitigating abnormal methylation that drives carcinogenesis (Qin et al. 2014).

#### Polyphenols

Several polyphenols act as DNMT inhibitors, reversing TSG hypermethylation and thus reactivating their functions. EGCG, derived from green tea, is among the most potent DNMT inhibitors, while quercetin, myricetin, and fisetin modulate DNA methylation by altering SAM ratios and inhibiting DNMTs (Duthie 2011a; Mikkelsen et al. 2021). Curcumin, in particular, inhibits DNMT1 activity, fostering hypomethylation and downregulating *DNMT1* mRNA in cancer cell studies (Al-Yousef et al. 2020; Andreescu et al. 2018; Chatterjee et al. 2019a). Additionally, curcumin interacts with DNMT1’s catalytic thiolate to target the enzyme directly, diminishing methylation in various cancer models (Liu et al. 2009).

#### Isothiocyanates

**Fig. 6:**
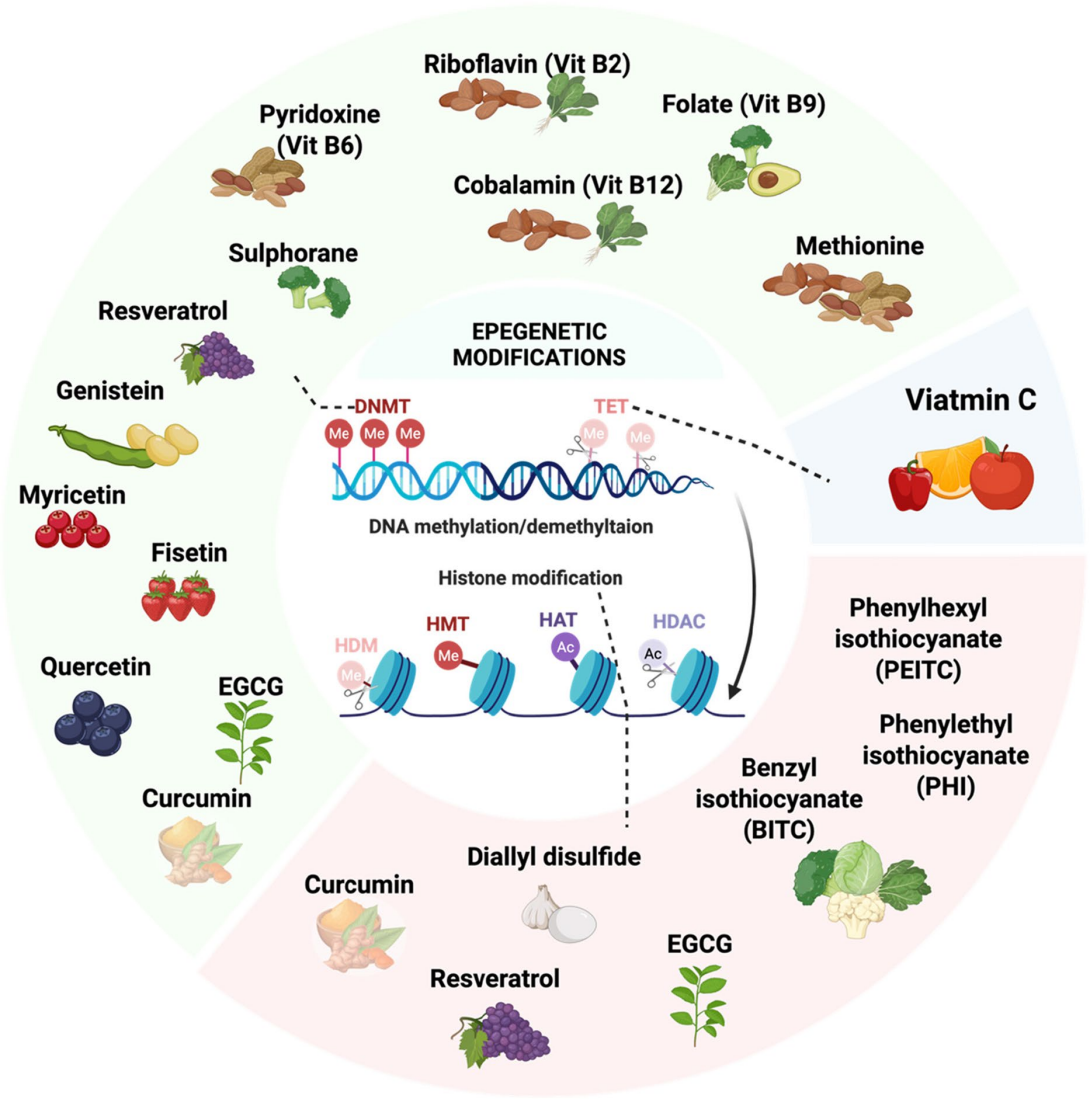
Global representation of different dietary ingredients with epigenetic modulations in cancer. The availability of nutrients, such as folate, methionine, cobalamin, pyridoxine, and riboflavin, which are involved in monocarbon metabolism, has been associated with cancer-related changes in DNA methylation. Furthermore, because they are potent inhibitors of DNMT activity, isothiocyanates and certain polyphenols, including quercetin, myricetin, and fisetin, can reverse DNA hypermethylation and reactivate TSG activity. The most potent inhibitor of DNMT is EGCG. Studies on epigenetic processes in cancer have shown that curcumin and resveratrol, as HAT inhibitors, control global histone acetylation. Also, it has been found that PEITC, BITC, and PHI can modify histones, which can inhibit the growth of many types of cancer. In addition, substances obtained from garlic, such as diallyl disulfide, suppress HDAC activity. In addition, by affecting the activity of the TET enzyme, vitamin C can control the expression of epigenetic genes. ***Abbreviations:*** TET, ten-eleven translocation methylcytosine dioxygenase; EGCG, epigallocatechin-3-gallate; PEITC, Phenethyl isothiocyanate; BITC, benzyl isothiocyanate; PHI, phenylhexyl isothiocyanate; DNMT, DNA methyltransferase; HDAC, histone deacetylase; HAT, histone acetyltransferase; HMT, histone methyltransferase

Isothiocyanates, including sulforaphane (SFN), modulate DNMT activity by downregulating DNMT1 and DNMT3a expression, particularly in breast and prostate cancer models (Hsu et al. 2011). SFN was also found to demethylate the promoter of *cyclin D2* (*CCND2*), enhancing its transcription and impeding DNMT-mediated silencing (Ho et al. 2011) (Fig. 6).

### Natural bioactive compounds as modulators of histone modifications

Curcumin is well-documented for its histone-modulating effects, acting as a HAT inhibitor and downregulating HDAC activities, which collectively help suppress tumor growth. Indeed, studies have shown curcumin’s ability to inhibit NF-κB acetylation, contributing to apoptosis induction and inhibition of cell proliferation in cancerous cells, such as lymphomas (Kang et al. 2005). In lymphoma cells specifically, curcumin has been found to downregulate HDAC1, HDAC3, and HDAC8 proteins, marking it as a potent anti-cancer agent targeting multiple HDAC enzymes (Ho et al. 2011). In medulloblastoma cells, the inhibition of HDAC4 by curcumin has additionally been linked to increased acetylation of tubulin, inducing cell cycle arrest and apoptosis (Lee et al. 2011). Further, it promotes histone H3K18 and H4K16 acetylation in MCF-7 breast cancer cells, a change associated with cell differentiation and reduced metastatic potential (Collins et al. 2013).

In cervical cancer, HDAC1 and HDAC2 are often overexpressed along with the viral oncoproteins E6 and E7 (Gao and Tollefsbol 2015). Curcumin treatment effectively reduces the expression of HDAC1/2, E6, and E7 proteins, which ultimately enhances the acetylation of tumor suppressor protein p53, enabling its reactivation (Gao and Tollefsbol 2015; Roy and Mukherjee 2014). Notably, in LNCaP prostate cancer cells, curcumin treatment resulted in a significant decline in global HDAC activity, possibly due to a reduction in HDAC8 expression. This treatment also decreased global levels of H3K27 trimethylation, a repressive epigenetic mark often elevated in cancers (Y. Gao and Tollefsbol 2015).

Resveratrol demonstrates multi-faceted anti-cancer properties through its modulation of SIRT1 and HDACs. In fact, this polyphenol upregulates SIRT1, a key regulator in apoptosis and cellular senescence, across various cancer types, enhancing cancer cell sensitivity to chemotherapy and inducing apoptosis (Li et al. 2013; Scuto et al. 2013). Furthermore, resveratrol has been observed to prevent the expression of class I, II, and IV HDACs in liver cancer cells (HepG2, Hep3B, and HuH7 cells), further inhibiting cancer cell growth and proliferation (Gao and Tollefsbol 2015; Venturelli et al. 2013).

SFN and other isothiocyanates (ITCs) target HDAC turnover and activity, increasing the acetylation of histone substrates and leading to TSG activation (Gao and Tollefsbol 2015). SFN treatment in HCT116 colorectal cancer cells is associated with increased HDAC release and upregulation of p21, a cyclin-dependent kinase inhibitor that induces cell cycle arrest (Myzak et al. 2006).

Ongoing treatment with SFN induces the release of HDAC3 from the 14–3-3 complex, facilitating its import into the nucleus to prevent degradation in the cytoplasm of colon cancer cells. Additionally, SFN has been shown to inhibit HDAC6 activity, thereby increasing heat shock protein 90 (Hsp90) acetylation, which may further modulate protein stability and function in cancer cells (Gibbs et al. 2009).

Various ITCs, such as phenylhexyl isothiocyanate (PHI), benzyl isothiocyanate (BITC), and phenethyl isothiocyanate (PEITC), exhibit potent histone-modulating effects, which can impede cancer development across multiple types. In fact, PHI administration in androgen-dependent LnCap cells leads to reduced HDAC activity, decreased HDAC1/2 expression, and increased levels of acetylated histones and p21 expression, indicating potential anti-proliferative effects (Beklemisheva et al. 2006; Ho et al. 2011).

PHI specifically inhibits the activity of HDAC1 and HDAC2 in LNCaP tumor cells, as demonstrated by Jiang et al. (Jiang et al. 2010). Furthermore, (Lu et al. 2008) observed that PHI induces hypomethylation of the *cyclin-dependant kinase inhibitor 2A* (*CDKN2A,* also known as *p16*) gene and hyperacetylation of histone H3 in myeloma cells, highlighting its dual impact on epigenetic modulation.

BITC treatment results in a significant reduction in both the activity and expression of HDAC1 and HDAC3 in pancreatic cancer cells (Gao and Tollefsbol 2015). The suppression of HDAC activity by BITC ultimately limits cancer cell growth, as confirmed by studies in pancreatic cancer models (Batra et al. 2010; Ho et al. 2011).

Through HDAC inhibition, EGCG effectively impedes invasive metastasis while simultaneously elevating H3 expression, indicating its potential role in limiting cancer progression (Bishop and Ferguson 2015; Kim & Kim 2013). Quercetin exerts anti-cancer effects by inhibiting p300 HAT activity, thereby downregulating NF-κB acetylation triggered by p300. Furthermore, quercetin significantly suppresses lysine-specific demethylase 1 (LSD1) activity, showcasing its capacity to inhibit histone demethylase activity, another critical epigenetic target in cancer cells (Gao and Tollefsbol 2015).

Compounds derived from garlic, such as diallyl sulfide (DAS), diallyl disulfide (DADS), and diallyl trisulfide (DATS), also demonstrate strong HDAC inhibitory properties. In prostate cancer cells, DADS treatment leads to increased apoptosis and histone acetylation, pointing to its apoptotic effects mediated through epigenetic regulation (Majid et al. 2008; Gao and Tollefsbol 2015).

DATS is metabolized to form allyl mercaptan (AM), which acts as a competitive HDAC inhibitor and exhibits actions similar to DADS (Ho et al. 2011). Notably, DADS, also referred to as 4,5-dithia-1,7-octadiene, has been shown to inhibit cell growth in colon cancer by suppressing HDAC activity, increasing the acetylation of histones H3 and H4, and downregulating the expression of *p21*^*waf1/cip1*^ (Majid et al. 2008; Gao and Tollefsbol 2015) (Fig. 6).

### Natural bioactive compounds as modulators of TET enzymes

Vitamin C plays a significant role in modulating the expression of epigenetic genes by influencing TET enzyme activity. When vitamin C is added to embryonic stem cell cultures, it results in a notable decrease in 5-mC levels and a moderate increase in 5-hmC, supporting a TET-dependent mechanism of DNA demethylation (Hore et al. 2016; Mastrangelo et al. 2018). Vitamin C enhances TET enzymatic activity by directly interacting with the catalytic domain of TET proteins. This interaction not only promotes proper TET folding but also improves Fe(II) recycling, which is essential for TET function (He et al. 2011; Hore et al. 2016; Mastrangelo et al. 2018; Mingay et al. 2018).

In leukemia models, vitamin C treatment has been shown to restore TET2 functionality, thereby inhibiting aberrant self-renewal and slowing leukemia progression (Cimmino et al. 2017). DNA demethylation effects were observed in large diffuse B-cell lymphoma and T-cell lymphoma cells treated with ascorbic acid (ASC), attributed to the induction of TET activity (Mastrangelo et al. 2018; Shenoy et al. 2017). Additionally, Fe(II) augments TET enzyme activity, and vitamin C further amplifies this effect when Fe(III) is present. Under alkaline conditions, Fe(II) can undergo spontaneous oxidation to Fe(III), creating an environment favorable for vitamin C-enhanced TET activity (Hore et al. 2016) (Fig. 6).

## Conclusion and perspectives

The landscape of cancer epigenetics continues to expand as diverse alterations in epigenetic pathways are increasingly discovered. The notion that cancer is not only a genetic but also an epigenetic disease has gained significant traction over the last decade. Genome-wide sequencing investigations have substantiated this view by revealing that numerous epigenetic regulators are frequently altered through both mutations and epimutations in cancer cells, leading to complex interactions that drive tumorigenesis. Mutations in genes that regulate epigenetic mechanisms have a pervasive impact on various aspects of cellular regulation, including chromatin remodeling, histone modifications, and DNA methylation.

While extensive research has elucidated many epigenetic alterations associated with cancer, the exact mechanisms by which these changes influence disease progression remain incompletely understood. Importantly, the dynamic and reversible nature of epigenetic modifications offers a promising avenue for disease prevention and therapeutic intervention. This review underscores the multifaceted roles of cancer epigenetics and highlights the potential of natural phytochemicals as anti-cancer agents. These compounds, derived from dietary sources, can modulate epigenetic pathways, promoting tumor cell apoptosis and inhibiting cell proliferation, invasion, metastasis, and cycle progression. Phytochemicals achieve these effects by activating TSGs while downregulating oncogenes, thus modulating gene and protein expression patterns implicated in cancer development and progression.

Extensive studies have shown that natural compounds, particularly flavonoids and polyphenols, influence epigenetic modifiers such as DNMTs, HDACs, and HMTs, as well as critical oncogenes and TSGs. Through these interactions, phytochemicals can sensitize cancer cells by targeting specific epigenetic mechanisms, thereby enhancing the efficacy of conventional chemotherapeutic agents. However, to maximize the therapeutic potential of these compounds, further research is needed to investigate their pharmacokinetics, particularly their metabolism and bioavailability, which remain insufficiently explored. Additionally, evaluating the toxicity of these phytochemicals is essential to ensure their safety and suitability for long-term use in cancer therapy.

Looking forward, research should focus on well-designed human clinical trials that examine the epigenetic effects of phytochemicals on cancer. These studies are critical for validating the clinical relevance of phytochemical-based therapies and could pave the way for personalized epigenetic diets aimed at cancer prevention and treatment. Ultimately, advancing our understanding of the epigenetic actions of phytochemicals could enable the development of more effective, personalized chemopreventive and therapeutic strategies tailored to individual epigenetic profiles in cancer management.

## Data Availability

No datasets were generated or analysed during the current study.
